# Supplementary material for: Application of Inverse Design Approaches to the Discovery of Nonlinear Optical Switches
Source: Molecules. 2023 Oct 31;28(21):7371. doi: 10.3390/molecules28217371 (PMC10647741; doi:10.3390/molecules28217371)
Supplement: Supplementary file 1 [file molecules-28-07371-s001.zip › molecules-2671407-supplementary.pdf]

The application of inverse design approaches to the discovery of nonlinear optical switches

Eline Desmedt, Léa Serrano Gimenez, Freija De Vleeschouwer,* and Mercedes Alonso*

Department of General Chemistry: Algemene Chemie (ALGC), Vrije Universiteit Brussel, Pleinlaan 2, 1050 Brussel, Belgium

E-mail: Freija.De.Vleeschouwer@vub.be; Mercedes.Alonso.Giner@vub.be

1 Equations related to the β_{HRS}

The HRS equations are written as Equation 1, under the condition that the laser's propagation plane is perpendicular to the incoherent scattered light.

$$\beta_{HRS}(-2\omega;\omega,\omega) = \sqrt{\langle\beta^2_{ZZZ}\rangle + \langle\beta^2_{ZXX}\rangle} \tag{1}$$

$\langle\beta^2_{ZZZ}\rangle$ and $\langle\beta^2_{ZXX}\rangle$ can be written as a function of the molecular components of the β tensor and their full expressions are written in Equations 2 and 3.

$$\begin{aligned}\langle\beta^2_{ZZZ}\rangle =& \frac{1}{7}\sum_{i}^{x,y,z}\beta^2_{iii} + \frac{4}{35}\sum_{i\neq j}^{x,y,z}\beta^2_{iij} + \frac{2}{35}\sum_{i\neq j}^{x,y,z}\beta_{iii}\beta_{ijj} + \frac{4}{35}\sum_{i\neq j}^{x,y,z}\beta_{jii}\beta_{iij} \\ &+\frac{4}{35}\sum_{i\neq j}^{x,y,z}\beta_{iii}\beta_{jji} + \frac{1}{35}\sum_{i}^{x,y,z}\beta^2_{jii} + \frac{4}{105}\sum_{i\neq j\neq k}^{x,y,z}\beta_{iij}\beta_{jkk} + \frac{1}{105}\sum_{i\neq j\neq k}^{x,y,z}\beta_{jii}\beta_{jkk} \\ &+\frac{4}{105}\sum_{i\neq j\neq k}^{x,y,z}\beta_{iij}\beta_{kkj} + \frac{2}{105}\sum_{i\neq j\neq k}^{x,y,z}\beta^2_{ijk} + \frac{4}{105}\sum_{i\neq j\neq k}^{x,y,z}\beta_{ijk}\beta_{jik}\end{aligned} \tag{2}$$

$$\begin{aligned}\langle\beta^2_{ZXX}\rangle =& \frac{1}{35}\sum_{i}^{x,y,z}\beta^2_{iii} + \frac{4}{105}\sum_{i\neq j}^{x,y,z}\beta_{iii}\beta_{ijj} - \frac{2}{35}\sum_{i\neq j}^{x,y,z}\beta_{iii}\beta_{jji} + \frac{8}{105}\sum_{i\neq j}^{x,y,z}\beta^2_{iij} \\ &+\frac{3}{35}\sum_{i\neq j}^{x,y,z}\beta^2_{ijj} - \frac{2}{35}\sum_{i}^{x,y,z}\beta_{ijj}\beta_{jii} + \frac{1}{35}\sum_{i\neq j\neq k}^{x,y,z}\beta_{ijj}\beta_{jkk} - \frac{2}{105}\sum_{i\neq j\neq k}^{x,y,z}\beta_{iik}\beta_{jjk} \\ &-\frac{2}{105}\sum_{i\neq j\neq k}^{x,y,z}\beta_{iij}\beta_{jkk} + \frac{2}{35}\sum_{i\neq j\neq k}^{x,y,z}\beta^2_{ijk} - \frac{2}{105}\sum_{i\neq j\neq k}^{x,y,z}\beta_{ijk}\beta_{jik}\end{aligned} \tag{3}$$

2 Computational Details

All quantum-chemical calculations were performed with the Gaussian16 software.[1] Our methodology relies mainly on density functional theory, where the CAM-B3LYP/6-311G(d,p) level of theory is employed for the geometry optimizations of the [26]-, [28]- and [30]-hexaphyrins with a Hückel topology.[2,3] To ensure that all optimized geometries are minima on the potential energy surface, we performed harmonic vibrational analyses at the same level of theory. Extensive benchmark studies on the energetics and kinetics of expanded porphyrins with both Hückel and Möbius topologies support the choice of the exchange-correlation functional.[4–6]

For this work, we focused on the HRS hyperpolarizabilities in static regime, which we evaluated with the coupled-perturbed Kohn–Sham equations to obtain the first hyperpolarizability related to the Hyper-Rayleigh Scattering (Equation 1). To compute these hyperpolarizabilities at a reasonable computational cost, we selected the CAM-B3LYP functional, which offers a semi-quantitative description of the NLO responses for expanded porphyrins with Hückel and Möbius topologies.[7–9] To describe the prevailing tensor components of the β_{HRS} with sufficient accuracy, at least a split valence double- or even triple-ζ basis sets with one set of diffuse and polarization functions is needed.[10–12] In addition, our choice of the CAM-B3LYP/6-311+G(d,p) level of theory relies on a recent benchmark study, where the influence of the amount of exact Hartree–Fock exchange included in the exchange-correlation functional on the magnitude of the static HRS responses was carefully investigated.[13]

Our in-house program CINDES was employed to carry out several inverse design procedures with the implemented best-first search (BFS) algorithm.[14,15] The fragment library contains 7 substituents for the *meso*-positions (NO_2, CN, F, H, CH_3, OH, and NH_2) and 4 heteroatoms for the core-modification sites (NH, O, S and Se), for which NH refers to the noncore-modified structure. CINDES generates the hexaphyrin structures and automatizes the quantum-chemical computations, *i.e.* geometry optimizations, harmonic vibrational analyses and NLO property calculations with the same level of theory as described above.

Each **28R**-hexaphyrin structure in database was converted to SMILES with the RDKit python library.[16] These SMILES were then converted to extended-connectivity fingerprints (ECFPs).[17] These fingerprints are represented by bit-vectors, for which the presence or absence of a certain part of the structure is encoded as a bit. Substructures are extracted from the main structure by starting from a non-hydrogen atom and extending the distance until two adjacent atoms are reached. These substructures are then transformed to 2048 bit-vectors. Similar to the Chemplot procedure, vectors containing only zeros or ones are discarded from the 2048 generated vectors.[18] Next, a dimension reduction is performed on the remaining generated bit-vectors with the T-distributed stochastic neighbor embedding,[19] as implemented in sci-kit learn python library[20] with a perplexity of 25 and Kullback-Leibler divergence of 0.817.

3 BFS procedures

3.1 26R → 28R

Table S1 : Global iteration structures of the BFS procedure on the maximization of the NLO contrast of the 28R ⇋ 26R switch with the X_Y_$R_{1,4}$_$R_{2,5}$_$R_{3,6}$ pattern. The static hyper-Rayleigh scattering first hyperpolarizability values of the [28]hexaphyrins and [26]hexaphyrins are given in a.u.

Global iteration 1	X_Y_$R_{1,4}$_$R_{2,5}$_$R_{3,6}$	β_{HRS}(28R)	β_{HRS}(26R)	NLO contrast
Substitution $R_{3,6}$	NH_O_NH_2_F_H	1.50×10^{-2}	1.01×10^{4}	1.01×10^{4}
	NH_O_NH_2_F_F	8.68×10^{-2}	1.34×10^{4}	1.34×10^{4}
	NH_O_NH_2_F_CN	1.17×10^{-1}	1.13×10^{4}	1.13×10^{4}
	NH_O_NH_2_F_NO_2	6.41	8.51×10^{3}	8.49×10^{3}
	NH_O_NH_2_F_CH_3	1.72×10^{-1}	1.14×10^{4}	1.14×10^{4}
	NH_O_NH_2_F_OH	2.29×10^{-1}	2.03×10^{4}	2.03×10^{4}
	NH_O_NH_2_F_NH_2	2.08	2.13×10^{4}	2.13×10^{4}
Substitution $R_{1,4}$	NH_O_H_F_NH_2	3.69×10^{-1}	7.77×10^{3}	7.77×10^{3}
	NH_O_F_F_NH_2	4.52×10^{-1}	1.15×10^{4}	1.15×10^{4}
	NH_O_CN_F_NH_2	1.96	7.68×10^{3}	7.67×10^{3}
	NH_O_NO_2_F_NH_2	1.27×10^{3}	7.85×10^{3}	4.75×10^{3}
	NH_O_CH_3_F_NH_2	5.22×10^{-1}	7.63×10^{3}	7.63×10^{3}
	NH_O_OH_F_NH_2	4.30	1.59×10^{4}	1.59×10^{4}
	NH_O_NH_2_F_NH_2	2.08	2.13×10^{4}	2.13×10^{4}
Core-modifications Y	NH_NH_NH_2_F_NH_2	2.01×10^{-1}	3.01×10^{4}	3.01×10^{4}
	NH_O_NH_2_F_NH_2	2.08	2.13×10^{4}	2.13×10^{4}
	NH_S_NH_2_F_NH_2	7.63×10^{-1}	2.81×10^{4}	2.80×10^{4}
	NH_Se_NH_2_F_NH_2	7.46×10^{-2}	2.98×10^{4}	2.98×10^{4}
Substitution $R_{2,5}$	NH_NH_NH_2_H_NH_2	8.92×10^{-1}	2.83×10^{4}	2.83×10^{4}
	NH_NH_NH_2_F_NH_2	2.01×10^{-1}	3.01×10^{4}	3.01×10^{4}
	NH_NH_NH_2_CN_NH_2	8.19×10^{-2}	5.99×10^{4}	5.99×10^{4}
	NH_NH_NH_2_NO_2_NH_2	5.76×10^{2}	5.21×10^{4}	5.04×10^{4}
	NH_NH_NH_2_CH_3_NH_2	1.12×10^{1}	3.31×10^{4}	3.31×10^{4}
	NH_NH_NH_2_OH_NH_2	1.94	3.24×10^{4}	3.24×10^{4}
	NH_NH_NH_2_NH_2_NH_2	2.72×10^{3}	2.27×10^{4}	1.57×10^{4}

Table S1 : Continuation - Global iteration structures of the BFS procedure on the maximization of the NLO contrast of the 28R $\leftrightharpoons$ 26R switch with the X_Y_R$_{1,4}$_R$_{2,5}$_R$_{3,6}$ pattern. The static hyper-Rayleigh scattering first hyperpolarizability values of the [28]hexaphyrins and [26]hexaphyrins are given in a.u.

Global iteration 2	X_Y_R$_{1,4}$_R$_{2,5}$_R$_{3,6}$	β_{HRS}(26R)	β_{HRS}(28R)	NLO contrast
	NH_NH_NH$_2$_H_NH$_2$	8.92×10^{-1}	2.83×10^{4}	2.83×10^{4}
	NH_NH_NH$_2$_F_NH$_2$	2.01×10^{-1}	3.01×10^{4}	3.01×10^{4}
	NH_NH_NH$_2$_CN_NH$_2$	8.19×10^{-2}	5.99×10^{4}	5.99×10^{4}
**Substitution R$_{2,5}$**	NH_NH_NH$_2$_NO$_2$_NH$_2$	5.76×10^{2}	5.21×10^{4}	5.04×10^{4}
	NH_NH_NH$_2$_CH$_3$_NH$_2$	1.12×10^{1}	3.31×10^{4}	3.31×10^{4}
	NH_NH_NH$_2$_OH_NH$_2$	1.94	3.24×10^{4}	3.24×10^{4}
	NH_NH_NH$_2$_NH$_2$_NH$_2$	2.72×10^{3}	2.27×10^{4}	1.57×10^{4}
	NH_NH_H_CN_NH$_2$	2.07×10^{-1}	2.47×10^{4}	2.47×10^{4}
	NH_NH_F_CN_NH$_2$	1.03×10^{-1}	3.35×10^{4}	3.35×10^{4}
	NH_NH_CN_CN_NH$_2$	2.29×10^{4}	7.75×10^{-1}	2.29×10^{4}
**Substitution R$_{1,4}$**	NH_NH_NO$_2$_CN_NH$_2$	2.92×10^{3}	2.43×10^{4}	1.68×10^{4}
	NH_NH_CH$_3$_CN_NH$_2$	5.00×10^{-2}	2.41×10^{4}	2.41×10^{4}
	NH_NH_OH_CN_NH$_2$	7.67×10^{-1}	4.28×10^{4}	4.28×10^{4}
	NH_NH_NH$_2$_CN_NH$_2$	8.19×10^{-2}	5.99×10^{4}	5.99×10^{4}
	NH_NH_NH$_2$_CN_H	4.92×10^{-2}	2.53×10^{4}	2.53×10^{4}
	NH_NH_NH$_2$_CN_F	7.26×10^{-1}	3.50×10^{4}	3.50×10^{4}
	NH_NH_NH$_2$_CN_CN	1.87×10^{-2}	2.07×10^{4}	2.07×10^{4}
**Substitution R$_{3,6}$**	NH_NH_NH$_2$_CN_NO$_2$	2.31	1.86×10^{4}	1.86×10^{4}
	NH_NH_NH$_2$_CN_CH$_3$	6.93×10^{-1}	2.67×10^{4}	2.67×10^{4}
	NH_NH_NH$_2$_CN_OH	1.35×10^{-1}	5.08×10^{4}	5.08×10^{4}
	NH_NH_NH$_2$_CN_NH$_2$	8.19×10^{-2}	5.99×10^{4}	5.99×10^{4}
	NH_NH_NH$_2$_CN_NH$_2$	8.19×10^{-2}	5.99×10^{4}	5.99×10^{4}
	NH_O_NH$_2$_CN_NH$_2$	1.12	3.84×10^{4}	3.84×10^{4}
**Core-modifications Y**	NH_S_NH$_2$_CN_NH$_2$	2.66	5.50×10^{4}	5.50×10^{4}
	NH_Se_NH$_2$_CN_NH$_2$	1.65	5.75×10^{4}	5.75×10^{4}

3.2 30R → 28R

Table S2 : Global iteration structures of the BFS procedure on the maximization of the NLO contrast of the 28R ⇋ 30R switch with the X_Y_$R_{1,4}$_$R_{2,5}$_$R_{3,6}$ pattern. The static hyper-Rayleigh scattering first hyperpolarizability values of the [28]hexaphyrins and [30]hexaphyrins are given in a.u.

Global iteration 1	X_Y_$R_{1,4}$_$R_{2,5}$_$R_{3,6}$	β_{HRS}(28R)	β_{HRS}(30R)	NLO contrast
	S_O_NH_2_F_H	1.35×10^3	3.46×10^3	9.22×10^2
	S_O_NH_2_F_F	1.24×10^3	2.98×10^3	7.16×10^2
	S_O_NH_2_F_CN	1.99×10^3	7.56×10^3	3.25×10^3
**Substitution $R_{3,6}$**	S_O_NH_2_F_NO_2	3.27×10^3	1.06×10^4	3.88×10^3
	S_O_NH_2_F_CH_3	1.55×10^3	3.14×10^3	5.38×10^2
	S_O_NH_2_F_OH	1.47×10^3	2.47×10^3	2.53×10^2
	S_O_NH_2_F_NH_2	1.57×10^3	2.65×10^3	2.76×10^2
	S_O_H_F_NO_2	1.23×10^3	5.06×10^3	2.33×10^3
	S_O_F_F_NO_2	1.63×10^3	5.79×10^3	2.33×10^3
	S_O_CN_F_NO_2	1.42×10^3	3.68×10^3	1.00×10^3
**Substitution $R_{1,4}$**	S_O_NO_2_F_NO_2	1.36×10^3	2.86×10^3	5.31×10^2
	S_O_CH_3_F_NO_2	1.67×10^3	5.39×10^3	1.97×10^3
	S_O_OH_F_NO_2	2.47×10^3	6.98×10^3	2.15×10^3
	S_O_NH_2_F_NO_2	3.27×10^3	1.06×10^4	3.88×10^3
	S_NH_NH_2_F_NO_2	2.89×10^3	2.91×10^3	7.20×10^{-2}
	S_O_NH_2_F_NO_2	3.27×10^3	1.06×10^4	3.88×10^3
**Core-modifications Y**	S_S_NH_2_F_NO_2	2.44×10^3	4.66×10^3	6.94×10^2
	S_Se_NH_2_F_NO_2	7.95×10^{-1}	4.08×10^3	4.08×10^3
	NH_Se_NH_2_F_NO_2	2.27×10^0	8.64×10^3	8.63×10^3
	O_Se_NH_2_F_NO_2	3.39×10^0	6.55×10^3	6.54×10^3
**Core-modifications X**	S_Se_NH_2_F_NO_2	7.95×10^{-1}	4.08×10^3	4.08×10^3
	Se_Se_NH_2_F_NO_2	0.00×10^0	3.77×10^3	3.77×10^3
	NH_Se_NH_2_H_NO_2	6.77×10^{-1}	8.95×10^3	8.94×10^3
	NH_Se_NH_2_F_NO_2	2.27×10^0	8.64×10^3	8.63×10^3
	NH_Se_NH_2_CN_NO_2	2.63×10^0	1.20×10^4	1.20×10^4
**Substitution $R_{2,5}$**	NH_Se_NH_2_NO_2_NO_2	1.86×10^2	6.20×10^3	5.67×10^3
	NH_Se_NH_2_CH_3_NO_2	2.88×10^{-1}	9.58×10^3	9.58×10^3
	NH_Se_NH_2_OH_NO_2	0.00×10^0	1.04×10^4	1.04×10^4
	NH_Se_NH_2_NH_2_NO_2	4.36×10^0	1.04×10^4	1.03×10^4

Table S2 : Continuation - Global iteration structures of the BFS procedure on the maximization of the NLO contrast of the 28R $\leftrightharpoons$ 30R switch with the X_Y_$R_{1,4}$_$R_{2,5}$_$R_{3,6}$ pattern. The static hyper-Rayleigh scattering first hyperpolarizability values of the [28]hexaphyrins and [30]hexaphyrins are given in a.u.

Global iteration 2	X_Y_$R_{1,4}$_$R_{2,5}$_$R_{3,6}$	β_{HRS}(28R)	β_{HRS}(30R)	NLO contrast
**Substitution $R_{2,5}$**	NH_Se_NH_2_H_NO_2	6.77×10^{-1}	8.95×10^{3}	8.94×10^{3}
	NH_Se_NH_2_F_NO_2	2.27×10^{0}	8.64×10^{3}	8.63×10^{3}
	NH_Se_NH_2_CN_NO_2	2.63×10^{0}	1.20×10^{4}	1.20×10^{4}
	NH_Se_NH_2_NO_2_NO_2	1.86×10^{2}	6.20×10^{3}	5.67×10^{3}
	NH_Se_NH_2_CH_3_NO_2	2.88×10^{-1}	9.58×10^{3}	9.58×10^{3}
	NH_Se_NH_2_OH_NO_2	0.00×10^{0}	1.04×10^{4}	1.04×10^{4}
	NH_Se_NH_2_NH_2_NO_2	4.36×10^{0}	1.04×10^{4}	1.03×10^{4}
**Substitution $R_{1,4}$**	NH_Se_H_CN_NO_2	3.27×10^{-1}	6.20×10^{3}	6.20×10^{3}
	NH_Se_F_CN_NO_2	7.92×10^{0}	7.42×10^{3}	7.39×10^{3}
	NH_Se_CN_CN_NO_2	8.32×10^{-1}	4.30×10^{3}	4.30×10^{3}
	NH_Se_NO_2_CN_NO_2	6.32×10^{2}	4.38×10^{3}	2.80×10^{3}
	NH_Se_CH_3_CN_NO_2	5.50×10^{-1}	7.20×10^{3}	7.20×10^{3}
	NH_Se_OH_CN_NO_2	7.66×10^{-1}	9.49×10^{3}	9.49×10^{3}
	NH_Se_NH_2_CN_NO_2	2.63×10^{0}	1.20×10^{4}	1.20×10^{4}
**Substitution $R_{3,6}$**	NH_Se_NH_2_CN_H	2.85×10^{-2}	5.81×10^{3}	5.81×10^{3}
	NH_Se_NH_2_CN_F	8.36×10^{0}	5.68×10^{3}	5.65×10^{3}
	NH_Se_NH_2_CN_CN	1.43×10^{0}	1.14×10^{4}	1.14×10^{4}
	NH_Se_NH_2_CN_NO_2	2.63×10^{0}	1.20×10^{4}	1.20×10^{4}
	NH_Se_NH_2_CN_CH_3	1.64×10^{-1}	5.67×10^{3}	5.67×10^{3}
	NH_Se_NH_2_CN_OH	5.19×10^{2}	6.07×10^{3}	4.67×10^{3}
	NH_Se_NH_2_CN_NH_2	1.65×10^{0}	7.11×10^{3}	7.11×10^{3}
**Core-modifications X**	NH_Se_NH_2_CN_NO_2	2.63×10^{0}	1.20×10^{4}	1.20×10^{4}
	O_Se_NH_2_CN_NO_2	1.06×10^{0}	1.12×10^{4}	1.12×10^{4}
	S_Se_NH_2_CN_NO_2	6.85×10^{2}	6.76×10^{3}	4.96×10^{3}
	Se_Se_NH_2_CN_NO_2	2.00×10^{3}	5.76×10^{3}	1.82×10^{3}
**Core-modifications Y**	NH_NH_NH_2_CN_NO_2	2.31×10^{0}	8.92×10^{3}	8.91×10^{3}
	NH_O_NH_2_CN_NO_2	3.96×10^{3}	1.24×10^{4}	4.34×10^{3}
	NH_S_NH_2_CN_NO_2	6.73×10^{-1}	1.35×10^{4}	1.35×10^{4}
	NH_Se_NH_2_CN_NO_2	2.63×10^{0}	1.20×10^{4}	1.20×10^{4}

Table S2 : Continuation - Global iteration structures of the BFS procedure on the maximization of the NLO contrast of the 28R ⇋ 30R switch with the X_Y_$R_{1,4}$_$R_{2,5}$_$R_{3,6}$ pattern. The static hyper-Rayleigh scattering first hyperpolarizability values of the [28]hexaphyrins and [30]hexaphyrins are given in a.u.

Global iteration 3	X_Y_$R_{1,4}$_$R_{2,5}$_$R_{3,6}$	β_{HRS}(28R)	β_{HRS}(30R)	NLO contrast
	NH_S_NH_2_H_NO_2	1.48×10^{-1}	1.00×10^{4}	1.00×10^{4}
	NH_S_NH_2_F_NO_2	2.25×10^{0}	9.70×10^{3}	9.69×10^{3}
	NH_S_NH_2_CN_NO_2	6.73×10^{-1}	1.35×10^{4}	1.35×10^{4}
**Substitution $R_{2,5}$**	NH_S_NH_2_NO_2_NO_2	0.00×10^{0}	6.40×10^{3}	6.40×10^{3}
	NH_S_NH_2_CH_3_NO_2	1.02×10^{-1}	1.06×10^{4}	1.06×10^{4}
	NH_S_NH_2_OH_NO_2	8.31×10^{2}	1.17×10^{4}	9.40×10^{3}
	NH_S_NH_2_NH_2_NO_2	4.58×10^{0}	1.11×10^{4}	1.11×10^{4}
	NH_S_NH_2_CN_H	4.31×10^{-2}	6.52×10^{3}	6.52×10^{3}
	NH_S_NH_2_CN_F	1.03×10^{1}	6.27×10^{3}	6.24×10^{3}
	NH_S_NH_2_CN_CN	1.42×10^{0}	1.35×10^{4}	1.35×10^{4}
**Substitution $R_{3,6}$**	NH_S_NH_2_CN_NO_2	6.73×10^{-1}	1.35×10^{4}	1.35×10^{4}
	NH_S_NH_2_CN_CH_3	8.02×10^{0}	6.37×10^{3}	6.34×10^{3}
	NH_S_NH_2_CN_OH	0.00×10^{0}	6.50×10^{3}	6.50×10^{3}
	NH_S_NH_2_CN_NH_2	2.66×10^{0}	7.80×10^{3}	7.79×10^{3}
	NH_S_H_CN_CN	7.42×10^{-2}	6.77×10^{3}	6.77×10^{3}
	NH_S_F_CN_CN	1.66×10^{0}	8.89×10^{3}	8.89×10^{3}
	NH_S_CN_CN_CN	6.06×10^{-1}	3.31×10^{3}	3.31×10^{3}
**Substitution $R_{3,6}$**	NH_S_NO_2_CN_CN	1.08×10^{3}	4.15×10^{3}	1.79×10^{3}
	NH_S_CH_3_CN_CN	2.15×10^{-1}	8.22×10^{3}	8.22×10^{3}
	NH_S_OH_CN_CN	0.00×10^{0}	1.20×10^{4}	1.20×10^{4}
	NH_S_NH_2_CN_CN	1.42×10^{0}	1.35×10^{4}	1.35×10^{4}
	NH_S_NH_2_CN_CN	1.42×10^{0}	1.35×10^{4}	1.35×10^{4}
	O_S_NH_2_CN_CN	3.36×10^{0}	1.72×10^{4}	1.72×10^{4}
**X**	S_S_NH_2_CN_CN	1.18×10^{3}	1.20×10^{4}	8.87×10^{3}
	Se_S_NH_2_CN_CN	1.54×10^{3}	1.10×10^{4}	7.13×10^{3}
	O_NH_NH_2_CN_CN	2.09×10^{0}	1.57×10^{4}	1.57×10^{4}
	O_O_NH_2_CN_CN	6.18×10^{-1}	2.08×10^{4}	2.08×10^{4}
**Y**	O_S_NH_2_CN_CN	3.36×10^{0}	1.72×10^{4}	1.72×10^{4}
	O_Se_NH_2_CN_CN	8.47×10^{-1}	1.34×10^{4}	1.34×10^{4}

Table S2 : Continuation - Global iteration structures of the BFS procedure on the maximization of the NLO contrast of the 28R ⇋ 30R switch with the X_Y_$R_{1,4}$_$R_{2,5}$_$R_{3,6}$ pattern. The static hyper-Rayleigh scattering first hyperpolarizability values of the [28]hexaphyrins and [30]hexaphyrins are given in a.u.

Global iteration 4	X_Y_$R_{1,4}$_$R_{2,5}$_$R_{3,6}$	β_{HRS}(28R)	β_{HRS}(30R)	NLO contrast
	O_O_NH_2_H_CN	2.20×10^{-2}	1.06×10^{4}	1.06×10^{4}
	O_O_NH_2_F_CN	1.19×10^{0}	1.00×10^{4}	1.00×10^{4}
	O_O_NH_2_CN_CN	6.18×10^{-1}	2.08×10^{4}	2.08×10^{4}
**Substitution $R_{2,5}$**	O_O_NH_2_NO_2_CN	1.98×10^{2}	1.72×10^{4}	1.66×10^{4}
	O_O_NH_2_CH_3_CN	1.18×10^{0}	9.86×10^{3}	9.86×10^{3}
	O_O_NH_2_OH_CN	2.31×10^{-1}	7.49×10^{3}	7.49×10^{3}
	O_O_NH_2_NH_2_CN	3.20×10^{2}	7.89×10^{3}	6.98×10^{3}
	NH_O_NH_2_CN_CN	6.66×10^{-1}	1.70×10^{4}	1.70×10^{4}
	O_O_NH_2_CN_CN	6.18×10^{-1}	2.08×10^{4}	2.08×10^{4}
**X**	S_O_NH_2_CN_CN	2.13×10^{3}	1.71×10^{4}	1.17×10^{4}
	Se_O_NH_2_CN_CN	2.17×10^{3}	1.39×10^{4}	8.55×10^{3}
	O_O_NH_2_CN_H	1.29×10^{-1}	1.07×10^{4}	1.07×10^{4}
	O_O_NH_2_CN_F	1.31×10^{0}	8.94×10^{3}	8.94×10^{3}
	O_O_NH_2_CN_CN	6.18×10^{-1}	2.08×10^{4}	2.08×10^{4}
**Substitution $R_{3,6}$**	O_O_NH_2_CN_NO_2	0.00×10^{0}	1.71×10^{4}	1.71×10^{4}
	O_O_NH_2_CN_CH_3	2.63×10^{-1}	8.84×10^{3}	8.84×10^{3}
	O_O_NH_2_CN_OH	2.79×10^{2}	6.43×10^{3}	5.64×10^{3}
	O_O_NH_2_CN_NH_2	3.99×10^{2}	7.47×10^{3}	6.36×10^{3}
	O_NH_NH_2_CN_CN	2.09×10^{0}	1.57×10^{4}	1.57×10^{4}
	O_O_NH_2_CN_CN	6.18×10^{-1}	2.08×10^{4}	2.08×10^{4}
**Y**	O_S_NH_2_CN_CN	3.36×10^{0}	1.72×10^{4}	1.72×10^{4}
	O_Se_NH_2_CN_CN	8.47×10^{-1}	1.34×10^{4}	1.34×10^{4}
	O_O_H_CN_CN	8.95×10^{-2}	7.16×10^{3}	7.16×10^{3}
	O_O_F_CN_CN	8.06×10^{-1}	9.04×10^{3}	9.04×10^{3}
	O_O_CN_CN_CN	1.00×10^{0}	3.07×10^{3}	3.07×10^{3}
**Substitution $R_{1,4}$**	O_O_NO_2_CN_CN	3.38×10^{2}	3.18×10^{3}	2.30×10^{3}
	O_O_CH_3_CN_CN	1.78×10^{-1}	8.08×10^{3}	8.07×10^{3}
	O_O_OH_CN_CN	1.19×10^{0}	1.61×10^{4}	1.61×10^{4}
	O_O_NH_2_CN_CN	6.18×10^{-1}	2.08×10^{4}	2.08×10^{4}

3.3 26R → 28R → 30R: starting point NH_NH_H_H_H

Table S3 : Global iteration structures of the BFS procedure on the maximization of the NLO contrast of the 26R → 28R → 30R switch with the X_Y_R$_{1,4}$_R$_{2,5}$_R$_{3,6}$ pattern. The static hyper-Rayleigh scattering first hyperpolarizability values of the [26]hexaphyrins, [28]hexaphyrins and [30]hexaphyrins are given in a.u.

Global iteration 1	X_Y_R$_{1,4}$_R$_{2,5}$_R$_{3,6}$	β_{HRS}(26R)	β_{HRS}(28R)	β_{HRS}(30R)	NLO contrast (26R)	NLO contrast (30R)	function
Substitution R$_{2,5}$	NH_NH_H_H_H	2.09×10^{3}	5.04×10^{-3}	2.18×10^{3}	2.09×10^{3}	2.18×10^{3}	2.04×10^{3}
	NH_NH_H_F_H	2.61×10^{3}	2.93×10^{-3}	1.61×10^{3}	2.61×10^{3}	1.61×10^{3}	1.30×10^{3}
	NH_NH_H_CN_H	5.73×10^{3}	4.62×10^{-4}	3.88×10^{3}	5.73×10^{3}	3.88×10^{3}	3.25×10^{3}
	NH_NH_H_NO$_2$_H	3.35×10^{3}	3.35×10^{2}	3.16×10^{3}	2.47×10^{3}	2.28×10^{3}	2.24×10^{3}
	NH_NH_H_CH$_3$_H	3.47×10^{3}	1.76×10^{-1}	1.79×10^{3}	3.47×10^{3}	1.79×10^{3}	1.36×10^{3}
	NH_NH_H_OH_H	2.91×10^{3}	1.55	1.35×10^{3}	2.91×10^{3}	1.35×10^{3}	9.86×10^{2}
	NH_NH_H_NH$_2$_H	2.49×10^{3}	1.64	1.32×10^{3}	2.48×10^{3}	1.31×10^{3}	1.01×10^{3}
Substitution R$_{3,6}$	NH_NH_H_CN_H	5.73×10^{3}	4.62×10^{-4}	3.88×10^{3}	5.73×10^{3}	3.88×10^{3}	3.25×10^{3}
	NH_NH_H_CN_F	9.20×10^{3}	0.00	4.53×10^{3}	9.20×10^{3}	4.53×10^{3}	3.38×10^{3}
	NH_NH_H_CN_CN	3.12×10^{3}	1.00×10^{-1}	6.72×10^{3}	3.12×10^{3}	6.72×10^{3}	2.28×10^{3}
	NH_NH_H_CN_NO$_2$	3.33×10^{3}	5.51×10^{-1}	7.48×10^{3}	3.33×10^{3}	7.47×10^{3}	2.41×10^{3}
	NH_NH_H_CN_CH$_3$	7.19×10^{3}	3.73	4.40×10^{3}	7.18×10^{3}	4.39×10^{3}	3.54×10^{3}
	NH_NH_H_CN_OH	1.80×10^{4}	3.94×10^{-1}	6.08×10^{3}	1.80×10^{4}	6.08×10^{3}	4.07×10^{3}
	NH_NH_H_CN_NH$_2$	2.47×10^{4}	2.07×10^{-1}	6.76×10^{3}	2.47×10^{4}	6.76×10^{3}	4.31×10^{3}
Substitution R$_{1,4}$	NH_NH_H_CN_NH$_2$	2.47×10^{4}	2.07×10^{-1}	6.76×10^{3}	2.47×10^{4}	6.76×10^{3}	4.31×10^{3}
	NH_NH_F_CN_NH$_2$	3.35×10^{4}	1.03×10^{-1}	6.29×10^{3}	3.35×10^{4}	6.29×10^{3}	3.74×10^{3}
	NH_NH_CN_CN_NH$_2$	2.29×10^{4}	7.75×10^{-1}	1.25×10^{4}	2.29×10^{4}	1.25×10^{4}	9.65×10^{3}
	NH_NH_NO$_2$_CN_NH$_2$	2.43×10^{4}	2.92×10^{3}	9.86×10^{3}	1.68×10^{4}	3.77×10^{3}	4.17×10^{3}
	NH_NH_CH$_3$_CN_NH$_2$	2.41×10^{4}	5.00×10^{-2}	6.51×10^{3}	2.41×10^{4}	6.51×10^{3}	4.13×10^{3}
	NH_NH_OH_CN_NH$_2$	4.28×10^{4}	7.67×10^{-1}	6.32×10^{3}	4.28×10^{4}	6.31×10^{3}	3.62×10^{3}
	NH_NH_NH$_2$_CN_NH$_2$	5.99×10^{4}	8.19×10^{-2}	6.75×10^{3}	5.99×10^{4}	6.75×10^{3}	3.76×10^{3}
Core-modifications Y	NH_NH_CN_CN_NH$_2$	2.29×10^{4}	7.75×10^{-1}	1.25×10^{4}	2.29×10^{4}	1.25×10^{4}	9.65×10^{3}
	NH_O_CN_CN_NH$_2$	1.42×10^{4}	6.57×10^{-1}	1.20×10^{4}	1.42×10^{4}	1.2×10^{4}	1.11×10^{4}
	NH_S_CN_CN_NH$_2$	2.14×10^{4}	2.77	1.36×10^{4}	2.14×10^{4}	1.36×10^{4}	1.11×10^{4}
	NH_Se_CN_CN_NH$_2$	2.19×10^{4}	1.38	1.29×10^{4}	2.19×10^{4}	1.29×10^{4}	1.03×10^{4}
**Global iteration 2**	**X_Y_R$_{1,4}$_R$_{2,5}$_R$_{3,6}$**	**β_{HRS}(26R)**	**β_{HRS}(28R)**	**β_{HRS}(30R)**	**NLO contrast (26R)**	**NLO contrast (30R)**	**function**
Substitution R$_{1,4}$	NH_S_H_CN_NH$_2$	2.05×10^{4}	2.53×10^{-2}	6.95×10^{3}	2.05×10^{4}	6.95×10^{3}	4.65×10^{3}
	NH_S_F_CN_NH$_2$	2.87×10^{4}	2.56	6.57×10^{3}	2.87×10^{4}	6.56×10^{3}	4.04×10^{3}
	NH_S_CN_CN_NH$_2$	2.14×10^{4}	2.77	1.36×10^{4}	2.14×10^{4}	1.36×10^{4}	1.11×10^{4}
	NH_S_NO$_2$_CN_NH$_2$	2.09×10^{4}	0.00	1.67×10^{4}	2.09×10^{4}	1.67×10^{4}	1.50×10^{4}
	NH_S_CH$_3$_CN_NH$_2$	2.03×10^{4}	1.02	6.77×10^{3}	2.03×10^{4}	6.76×10^{3}	4.51×10^{3}
	NH_S_OH_CN_NH$_2$	3.74×10^{4}	4.34×10^{-1}	7.32×10^{3}	3.74×10^{4}	7.32×10^{3}	4.38×10^{3}
	NH_S_NH$_2$_CN_NH$_2$	5.50×10^{4}	2.66	7.80×10^{3}	5.50×10^{4}	7.79×10^{3}	4.45×10^{3}
Core-modifications Y	NH_NH_NO$_2$_CN_NH$_2$	2.43×10^{4}	2.92×10^{3}	9.86×10^{3}	1.68×10^{4}	3.77×10^{3}	4.17×10^{3}
	NH_O_NO$_2$_CN_NH$_2$	1.46×10^{4}	2.80×10^{3}	7.47×10^{3}	8.03×10^{3}	2.12×10^{3}	2.59×10^{3}
	NH_S_NO$_2$_CN_NH$_2$	2.09×10^{4}	0.00	1.67×10^{4}	2.09×10^{4}	1.67×10^{4}	1.50×10^{4}
	NH_Se_NO$_2$_CN_NH$_2$	2.12×10^{4}	1.46	1.61×10^{4}	2.12×10^{4}	1.61×10^{4}	1.42×10^{4}
Substitution R$_{3,6}$	NH_S_NO$_2$_CN_H	2.98×10^{3}	6.50×10^{2}	8.94×10^{3}	1.49×10^{3}	7.17×10^{3}	1.44×10^{3}
	NH_S_NO$_2$_CN_F	5.90×10^{3}	6.73×10^{2}	1.07×10^{4}	4.16×10^{3}	8.83×10^{3}	3.59×10^{3}
	NH_S_NO$_2$_CN_CN	8.24×10^{2}	1.08×10^{3}	4.15×10^{3}	3.56×10^{1}	1.79×10^{3}	1.82×10^{2}
	NH_S_NO$_2$_CN_NO$_2$	1.29×10^{3}	6.26×10^{2}	4.38×10^{3}	2.29×10^{2}	2.81×10^{3}	4.47×10^{2}
	NH_S_NO$_2$_CN_CH$_3$	4.37×10^{3}	1.72×10^{3}	8.99×10^{3}	1.16×10^{3}	4.94×10^{3}	1.48×10^{3}
	NH_S_NO$_2$_CN_OH	1.36×10^{4}	0.00	1.28×10^{4}	1.36×10^{4}	1.28×10^{4}	1.24×10^{4}
	NH_S_NO$_2$_CN_NH$_2$	2.09×10^{4}	0.00	1.67×10^{4}	2.09×10^{4}	1.67×10^{4}	1.50×10^{4}
Substitution R$_{2,5}$	NH_S_NO$_2$_H_NH$_2$	1.02×10^{4}	3.44×10^{-1}	1.45×10^{4}	1.02×10^{4}	1.45×10^{4}	8.73×10^{3}
	NH_S_NO$_2$_F_NH$_2$	1.12×10^{4}	2.27	1.34×10^{4}	1.12×10^{4}	1.34×10^{4}	1.02×10^{4}
	NH_S_NO$_2$_CN_NH$_2$	2.09×10^{4}	0.00	1.67×10^{4}	2.09×10^{4}	1.67×10^{4}	1.50×10^{4}
	NH_S_NO$_2$_NO$_2$_NH$_2$	1.60×10^{4}	1.14×10^{3}	1.44×10^{4}	1.29×10^{4}	1.13×10^{4}	1.09×10^{4}
	NH_S_NO$_2$_CH$_3$_NH$_2$	1.28×10^{4}	1.18	1.34×10^{4}	1.28×10^{4}	1.34×10^{4}	1.25×10^{4}
	NH_S_NO$_2$_OH_NH$_2$	1.28×10^{4}	5.41×10^{2}	1.46×10^{4}	1.12×10^{4}	1.31×10^{4}	1.06×10^{4}
	NH_S_NO$_2$_NH$_2$_NH$_2$	1.06×10^{4}	0.00	1.44×10^{4}	1.06×10^{4}	1.44×10^{4}	9.18×10^{3}

3.4 26R → 28R → 30R: starting point NH_NH_NH_2_CN_NH_2

Table S4 : Global iteration structures of the BFS procedure on the maximization of the NLO contrast of the 26R → 28R → 30R switch with the X_Y_$R_{1,4}$_$R_{2,5}$_$R_{3,6}$ pattern. The static hyper-Rayleigh scattering first hyperpolarizability values of the [26]hexaphyrins, [28]hexaphyrins and [30]hexaphyrins are given in a.u.

Global iteration 1	X_Y_$R_{1,4}$_$R_{2,5}$_$R_{3,6}$	β_{HRS}(26R)	β_{HRS}(28R)	β_{HRS}(30R)	NLO contrast (26R)	NLO contrast (30R)	function
**Substitution $R_{1,4}$**	NH_NH_H_CN_NH_2	2.47×10^{4}	2.07×10^{-1}	6.76×10^{3}	2.47×10^{4}	6.76×10^{3}	4.31×10^{3}
	NH_NH_F_CN_NH_2	3.35×10^{4}	1.03×10^{-1}	6.29×10^{3}	3.35×10^{4}	6.29×10^{3}	3.74×10^{3}
	NH_NH_CN_CN_NH_2	2.29×10^{4}	7.75×10^{-1}	1.25×10^{4}	2.29×10^{4}	1.25×10^{4}	9.65×10^{3}
	NH_NH_NO_2_CN_NH_2	2.43×10^{4}	2.92×10^{3}	9.86×10^{3}	1.68×10^{4}	3.77×10^{3}	4.17×10^{3}
	NH_NH_CH_3_CN_NH_2	2.41×10^{4}	5.00×10^{-2}	6.51×10^{3}	2.41×10^{4}	6.51×10^{3}	4.13×10^{3}
	NH_NH_OH_CN_NH_2	4.28×10^{4}	7.67×10^{-1}	6.32×10^{3}	4.28×10^{4}	6.31×10^{3}	3.62×10^{3}
	NH_NH_NH_2_CN_NH_2	5.99×10^{4}	8.19×10^{-2}	6.75×10^{3}	5.99×10^{4}	6.75×10^{3}	3.76×10^{3}
**Core-modifications Y**	NH_NH_CN_CN_NH_2	2.29×10^{4}	7.75×10^{-1}	1.25×10^{4}	2.29×10^{4}	1.25×10^{4}	9.65×10^{3}
	NH_O_CN_CN_NH_2	1.42×10^{4}	6.57×10^{-1}	1.20×10^{4}	1.42×10^{4}	1.20×10^{4}	1.11×10^{4}
	NH_S_CN_CN_NH_2	2.14×10^{4}	2.77	1.36×10^{4}	2.14×10^{4}	1.36×10^{4}	1.11×10^{4}
	NH_Se_CN_CN_NH_2	2.19×10^{4}	1.38	1.29×10^{4}	2.19×10^{4}	1.29×10^{4}	1.03×10^{4}
**Substitution $R_{3,6}$**	NH_S_CN_CN_H	2.97×10^{3}	2.83×10^{-2}	6.82×10^{3}	2.97×10^{3}	6.82×10^{3}	2.13×10^{3}
	NH_S_CN_CN_F	6.06×10^{3}	1.17	8.8×10^{3}	6.06×10^{3}	8.8×10^{3}	5.11×10^{3}
	NH_S_CN_CN_CN	735	6.06×10^{-1}	3.31×10^{3}	7.33×10^{2}	3.31×10^{3}	4.49×10^{2}
	NH_S_CN_CN_NO_2	1.46×10^{3}	8.96×10^{-1}	4.33×10^{3}	1.46×10^{3}	4.33×10^{3}	9.77×10^{2}
	NH_S_CN_CN_CH_3	4.38×10^{3}	6.49×10^{-1}	7.80×10^{3}	4.38×10^{3}	7.79×10^{3}	3.42×10^{3}
	NH_S_CN_CN_OH	1.4×10^{4}	5.57×10^{-1}	1.25×10^{4}	1.40×10^{4}	1.25×10^{4}	1.18×10^{4}
	NH_S_CN_CN_NH_2	2.14×10^{4}	2.77	1.36×10^{4}	2.14×10^{4}	1.36×10^{4}	1.11×10^{4}
**Substitution $R_{2,5}$**	NH_S_CN_H_OH	7.55×10^{3}	3.83×10^{-1}	9.11×10^{3}	7.55×10^{3}	9.11×10^{3}	6.91×10^{3}
	NH_S_CN_F_OH	8.4×10^{3}	1.01×10^{-1}	8.33×10^{3}	8.40×10^{3}	8.33×10^{3}	8.29×10^{3}
	NH_S_CN_CN_OH	1.4×10^{4}	5.57×10^{-1}	1.25×10^{4}	1.40×10^{4}	1.25×10^{4}	1.18×10^{4}
	NH_S_CN_NO_2_OH	1.03×10^{4}	1.38×10^{2}	1.19×10^{4}	9.91×10^{3}	1.14×10^{4}	9.29×10^{3}
	NH_S_CN_CH_3_OH	9.65×10^{3}	3.03×10^{-1}	8.79×10^{3}	9.65×10^{3}	8.79×10^{3}	8.40×10^{3}
	NH_S_CN_OH_OH	1.04×10^{4}	5.51×10^{-1}	8.51×10^{3}	1.04×10^{4}	8.50×10^{3}	7.73×10^{3}
	NH_S_CN_NH_2_OH	1.17×10^{4}	9.62×10^{-1}	8.93×10^{3}	1.17×10^{4}	8.93×10^{3}	7.87×10^{3}
**Global iteration 2**	**X_Y_$R_{1,4}$_$R_{2,5}$_$R_{3,6}$**	**β_{HRS}(26R)**	**β_{HRS}(28R)**	**β_{HRS}(30R)**	**NLO contrast (26R)**	**NLO contrast (30R)**	**function**
**Core-modifications Y**	NH_NH_CN_CN_OH	1.57×10^{4}	7.77×10^{-1}	1.09×10^{4}	1.57×10^{4}	1.09×10^{4}	9.30×10^{3}
	NH_O_CN_CN_OH	9.83×10^{3}	0.00	9.71×10^{3}	9.83×10^{3}	9.71×10^{3}	9.66×10^{3}
	NH_S_CN_CN_OH	1.40×10^{4}	5.57×10^{-1}	1.25×10^{4}	1.40×10^{4}	1.25×10^{4}	1.18×10^{4}
	NH_Se_CN_CN_OH	1.41×10^{4}	1.43	1.17×10^{4}	1.41×10^{4}	1.17×10^{4}	1.07×10^{4}
**Substitution $R_{3,6}$**	NH_S_CN_CN_H	2.97×10^{3}	2.83×10^{-2}	6.82×10^{3}	2.97×10^{3}	6.82×10^{3}	2.13×10^{3}
	NH_S_CN_CN_F	6.06×10^{3}	1.17	8.80×10^{3}	6.06×10^{3}	8.80×10^{3}	5.11×10^{3}
	NH_S_CN_CN_CN	7.35×10^{2}	6.06×10^{-1}	3.31×10^{3}	7.33×10^{2}	3.31×10^{3}	4.49×10^{2}
	NH_S_CN_CN_NO_2	1.46×10^{3}	8.96×10^{-1}	4.33×10^{3}	1.46×10^{3}	4.33×10^{3}	9.77×10^{2}
	NH_S_CN_CN_CH_3	4.38×10^{3}	6.49×10^{-1}	7.80×10^{3}	4.38×10^{3}	7.79×10^{3}	3.42×10^{3}
	NH_S_CN_CN_OH	1.40×10^{4}	5.57×10^{-1}	1.25×10^{4}	1.4×10^{4}	1.25×10^{4}	1.18×10^{4}
	NH_S_CN_CN_NH_2	2.14×10^{4}	2.77	1.36×10^{4}	2.14×10^{4}	1.36×10^{4}	1.11×10^{4}
**Substitution $R_{2,5}$**	NH_S_CN_H_OH	7.55×10^{3}	3.83×10^{-1}	9.11×10^{3}	7.55×10^{3}	9.11×10^{3}	6.91×10^{3}
	NH_S_CN_F_OH	8.40×10^{3}	1.01×10^{-1}	8.33×10^{3}	8.40×10^{3}	8.33×10^{3}	8.29×10^{3}
	NH_S_CN_CN_OH	1.40×10^{4}	5.57×10^{-1}	1.25×10^{4}	1.40×10^{4}	1.25×10^{4}	1.18×10^{4}
	NH_S_CN_NO_2_OH	1.03×10^{4}	1.38×10^{2}	1.19×10^{4}	9.91×10^{3}	1.14×10^{4}	9.29×10^{3}
	NH_S_CN_CH_3_OH	9.65×10^{3}	3.03×10^{-1}	8.79×10^{3}	9.65×10^{3}	8.79×10^{3}	8.40×10^{3}
	NH_S_CN_OH_OH	1.04×10^{4}	5.51×10^{-1}	8.51×10^{3}	1.04×10^{4}	8.50×10^{3}	7.73×10^{3}
	NH_S_CN_NH_2_OH	1.17×10^{4}	9.62×10^{-1}	8.93×10^{3}	1.17×10^{4}	8.93×10^{3}	7.87×10^{3}
**Substitution $R_{1,4}$**	NH_S_H_CN_OH	1.44×10^{4}	9.89×10^{-1}	6.75×10^{3}	1.44×10^{4}	6.75×10^{3}	4.95×10^{3}
	NH_S_F_CN_OH	2.03×10^{4}	4.38×10^{-1}	6.05×10^{3}	2.03×10^{4}	6.04×10^{3}	3.92×10^{3}
	NH_S_CN_CN_OH	1.40×10^{4}	5.57×10^{-1}	1.25×10^{4}	1.40×10^{4}	1.25×10^{4}	1.18×10^{4}
	NH_S_NO_2_CN_OH	1.36×10^{4}	0.00	1.28×10^{4}	1.36×10^{4}	1.28×10^{4}	1.24×10^{4}
	NH_S_CH_3_CN_OH	1.51×10^{4}	6.60×10^{-2}	6.62×10^{3}	1.51×10^{4}	6.62×10^{3}	4.75×10^{3}
	NH_S_OH_CN_OH	2.77×10^{4}	1.23×10^{-1}	6.54×10^{3}	2.77×10^{4}	6.54×10^{3}	4.05×10^{3}
	NH_S_NH_2_CN_OH	4.32×10^{4}	0.00	6.50×10^{3}	4.32×10^{4}	6.50×10^{3}	3.74×10^{3}
**Global iteration 3**	**X_Y_$R_{1,4}$_$R_{2,5}$_$R_{3,6}$**	**β_{HRS}(26R)**	**β_{HRS}(28R)**	**β_{HRS}(30R)**	**NLO contrast (26R)**	**NLO contrast (30R)**	**function**
**Substitution $R_{2,5}$**	NH_S_NO_2_H_OH	7.15×10^{3}	1.14×10^{3}	1.11×10^{4}	4.35×10^{3}	8.08×10^{3}	4.01×10^{3}
	NH_S_NO_2_F_OH	7.97×10^{3}	1.02×10^{3}	1.00×10^{4}	5.37×10^{3}	7.32×10^{3}	5.06×10^{3}
	NH_S_NO_2_CN_OH	1.36×10^{4}	0.00	1.28×10^{4}	1.36×10^{4}	1.28×10^{4}	1.24×10^{4}
	NH_S_NO_2_NO_2_OH	9.90×10^{3}	1.85×10^{3}	1.19×10^{4}	5.51×10^{3}	7.31×10^{3}	5.35×10^{3}
	NH_S_NO_2_CH_3_OH	9.33×10^{3}	1.30×10^{3}	1.04×10^{4}	6.06×10^{3}	7.09×10^{3}	5.89×10^{3}
	NH_S_NO_2_OH_OH	9.61×10^{3}	9.22×10^{2}	1.08×10^{4}	7.17×10^{3}	8.35×10^{3}	6.88×10^{3}
	NH_S_NO_2_NH_2_OH	1.02×10^{4}	9.73×10^{2}	1.00×10^{4}	7.63×10^{3}	7.44×10^{3}	7.39×10^{3}

Table S4 : Continuation - Global iteration structures of the BFS procedure on the maximization of the NLO contrast of the 26R → 28R → 30R switch with the X_Y_R$_{1,4}$_R$_{2,5}$_R$_{3,6}$ pattern. The static hyper-Rayleigh scattering first hyperpolarizability values of the [26]hexaphyrins, [28]hexaphyrins and [30]hexaphyrins are given in a.u.

Global iteration 3	X_Y_R$_{1,4}$_R$_{2,5}$_R$_{3,6}$	β_{HRS}(26R)	β_{HRS}(28R)	β_{HRS}(30R)	NLO contrast (26R)	NLO contrast (30R)	function
Substitution R$_{3,6}$	NH_S_NO$_2$_CN_H	2.98×10^{3}	6.50×10^{2}	8.94×10^{3}	1.49×10^{3}	7.17×10^{3}	1.44×10^{3}
	NH_S_NO$_2$_CN_F	5.90×10^{3}	6.73×10^{2}	1.07×10^{4}	4.16×10^{3}	8.83×10^{3}	3.59×10^{3}
	NH_S_NO$_2$_CN_CN	8.24×10^{2}	1.08×10^{3}	4.15×10^{3}	3.56×10^{1}	1.79×10^{3}	1.82×10^{2}
	NH_S_NO$_2$_CN_NO$_2$	1.29×10^{3}	6.26×10^{2}	4.38×10^{3}	2.29×10^{2}	2.81×10^{3}	4.47×10^{2}
	NH_S_NO$_2$_CN_CH$_3$	4.37×10^{3}	1.72×10^{3}	8.99×10^{3}	1.16×10^{3}	4.94×10^{3}	1.48×10^{3}
	NH_S_NO$_2$_CN_OH	1.36×10^{4}	0.00	1.28×10^{4}	1.36×10^{4}	1.28×10^{4}	1.24×10^{4}
	NH_S_NO$_2$_CN_NH$_2$	2.09×10^{4}	0.00	1.67×10^{4}	2.09×10^{4}	1.67×10^{4}	1.50×10^{4}
Core-modifications Y	NH_NH_NO$_2$_CN_NH$_2$	2.43×10^{4}	2.92×10^{3}	9.86×10^{3}	1.68×10^{4}	3.77×10^{3}	4.17×10^{3}
	NH_O_NO$_2$_CN_NH$_2$	1.46×10^{4}	2.80×10^{3}	7.47×10^{3}	8.03×10^{3}	2.12×10^{3}	2.59×10^{3}
	NH_S_NO$_2$_CN_NH$_2$	2.09×10^{4}	0.00	1.67×10^{4}	2.09×10^{4}	1.67×10^{4}	1.50×10^{4}
	NH_Se_NO$_2$_CN_NH$_2$	2.12×10^{4}	1.46	1.61×10^{4}	2.12×10^{4}	1.61×10^{4}	1.42×10^{4}
Substitution R$_{1,4}$	NH_S_H_CN_NH$_2$	2.05×10^{4}	2.53×10^{-2}	6.95×10^{3}	2.05×10^{4}	6.95×10^{3}	4.65×10^{3}
	NH_S_F_CN_NH$_2$	2.87×10^{4}	2.56	6.57×10^{3}	2.87×10^{4}	6.56×10^{3}	4.04×10^{3}
	NH_S_CN_CN_NH$_2$	2.14×10^{4}	2.77	1.36×10^{4}	2.14×10^{4}	1.36×10^{4}	1.11×10^{4}
	NH_S_NO$_2$_CN_NH$_2$	2.09×10^{4}	0.00	1.67×10^{4}	2.09×10^{4}	1.67×10^{4}	1.50×10^{4}
	NH_S_CH$_3$_CN_NH$_2$	2.03×10^{4}	1.02	6.77×10^{3}	2.03×10^{4}	6.76×10^{3}	4.51×10^{3}
	NH_S_OH_CN_NH$_2$	3.74×10^{4}	4.34×10^{-1}	7.32×10^{3}	3.74×10^{4}	7.32×10^{3}	4.38×10^{3}
	NH_S_NH$_2$_CN_NH$_2$	5.50×10^{4}	2.66	7.80×10^{3}	5.50×10^{4}	7.79×10^{3}	4.45×10^{3}
**Global iteration 4**	**X_Y_R$_{1,4}$_R$_{2,5}$_R$_{3,6}$**	**β_{HRS}(26R)**	**β_{HRS}(28R)**	**β_{HRS}(30R)**	**NLO contrast (26R)**	**NLO contrast (30R)**	**function**
Core-modifications Y	NH_NH_NO$_2$_CN_NH$_2$	2.43×10^{4}	2.92×10^{3}	9.86×10^{3}	1.68×10^{4}	3.77×10^{3}	4.17×10^{3}
	NH_O_NO$_2$_CN_NH$_2$	1.46×10^{4}	2.80×10^{3}	7.47×10^{3}	8.03×10^{3}	2.12×10^{3}	2.59×10^{3}
	NH_S_NO$_2$_CN_NH$_2$	2.09×10^{4}	0.00	1.67×10^{4}	2.09×10^{4}	1.67×10^{4}	1.50×10^{4}
	NH_Se_NO$_2$_CN_NH$_2$	2.12×10^{4}	1.46	1.61×10^{4}	2.12×10^{4}	1.61×10^{4}	1.42×10^{4}
Substitution R$_{2,5}$	NH_S_NO$_2$_H_NH$_2$	1.02×10^{4}	3.44×10^{-1}	1.45×10^{4}	1.02×10^{4}	1.45×10^{4}	8.73×10^{3}
	NH_S_NO$_2$_F_NH$_2$	1.12×10^{4}	2.27	1.34×10^{4}	1.12×10^{4}	1.34×10^{4}	1.02×10^{4}
	NH_S_NO$_2$_CN_NH$_2$	2.09×10^{4}	0.00	1.67×10^{4}	2.09×10^{4}	1.67×10^{4}	1.50×10^{4}
	NH_S_NO$_2$_NO$_2$_NH$_2$	1.60×10^{4}	1.14×10^{3}	1.44×10^{4}	1.29×10^{4}	1.13×10^{4}	1.09×10^{4}
	NH_S_NO$_2$_CH$_3$_NH$_2$	1.28×10^{4}	1.18	1.34×10^{4}	1.28×10^{4}	1.34×10^{4}	1.25×10^{4}
	NH_S_NO$_2$_OH_NH$_2$	1.28×10^{4}	5.41×10^{2}	1.46×10^{4}	1.12×10^{4}	1.31×10^{4}	1.06×10^{4}
	NH_S_NO$_2$_NH$_2$_NH$_2$	1.06×10^{4}	0.00	1.44×10^{4}	1.06×10^{4}	1.44×10^{4}	9.18×10^{3}
Substitution R$_{1,4}$	NH_S_H_CN_NH$_2$	2.05×10^{4}	2.53×10^{-2}	6.95×10^{3}	2.05×10^{4}	6.95×10^{3}	4.65×10^{3}
	NH_S_F_CN_NH$_2$	2.87×10^{4}	2.56	6.57×10^{3}	2.87×10^{4}	6.56×10^{3}	4.04×10^{3}
	NH_S_CN_CN_NH$_2$	2.14×10^{4}	2.77	1.36×10^{4}	2.14×10^{4}	1.36×10^{4}	1.11×10^{4}
	NH_S_NO$_2$_CN_NH$_2$	2.09×10^{4}	0.00	1.67×10^{4}	2.09×10^{4}	1.67×10^{4}	1.50×10^{4}
	NH_S_CH$_3$_CN_NH$_2$	2.03×10^{4}	1.02	6.77×10^{3}	2.03×10^{4}	6.76×10^{3}	4.51×10^{3}
	NH_S_OH_CN_NH$_2$	3.74×10^{4}	4.34×10^{-1}	7.32×10^{3}	3.74×10^{4}	7.32×10^{3}	4.38×10^{3}
	NH_S_NH$_2$_CN_NH$_2$	5.50×10^{4}	2.66	7.80×10^{3}	5.50×10^{4}	7.79×10^{3}	4.45×10^{3}
Substitution R$_{3,6}$	NH_S_NO$_2$_CN_H	2.98×10^{3}	6.50×10^{2}	8.94×10^{3}	1.49×10^{3}	7.17×10^{3}	1.44×10^{3}
	NH_S_NO$_2$_CN_F	5.90×10^{3}	6.73×10^{2}	1.07×10^{4}	4.16×10^{3}	8.83×10^{3}	3.59×10^{3}
	NH_S_NO$_2$_CN_CN	8.24×10^{2}	1.08×10^{3}	4.15×10^{3}	3.56×10^{1}	1.79×10^{3}	1.82×10^{2}
	NH_S_NO$_2$_CN_NO$_2$	1.29×10^{3}	6.26×10^{2}	4.38×10^{3}	2.29×10^{2}	2.81×10^{3}	4.47×10^{2}
	NH_S_NO$_2$_CN_CH$_3$	4.37×10^{3}	1.72×10^{3}	8.99×10^{3}	1.16×10^{3}	4.94×10^{3}	1.48×10^{3}
	NH_S_NO$_2$_CN_OH	1.36×10^{4}	0.00	1.28×10^{4}	1.36×10^{4}	1.28×10^{4}	1.24×10^{4}
	NH_S_NO$_2$_CN_NH$_2$	2.09×10^{4}	0.00	1.67×10^{4}	2.09×10^{4}	1.67×10^{4}	1.50×10^{4}

4 Datasets comparison: 26R $\rightarrow$ 28R *versus* 30R $\rightarrow$ 28R

In the main manuscript, we established that the two redox switches have their own ideal recipe to maximize the NLO contrast. In an attempt to generalize our findings, a detailed analysis of the generated databases for both switches is carried out to derive guiding principles for high-potential hexaphyrin-based NLO switches. From our previous studies[21,22] and the BFS runs performed in this work, we collected in total 242 and 320 different functionalization patterns for the **26R** $\rightarrow$ **28R** and **30R** $\rightarrow$ **28R** switches, respectively. The distribution of the NLO contrast values over different ranges is presented in the pie charts in Figure 1A and 1B for each type of redox switch. About 64% of the **26R** $\rightarrow$ **28R** molecular switches have contrast values below 15000 a.u., whereas the majority of the functionalized **30R** $\rightarrow$ **28R** molecular switches is found within the 4000-8000 a.u. range. Note that for both the **26R** and **30R**-based switches only a select group of 8% and 4% has contrasts larger than 30000 a.u. and 16000 a.u., respectively. Of course, the two BFS optima retrieved in Section **??** fall within these high-NLO contrast groups.

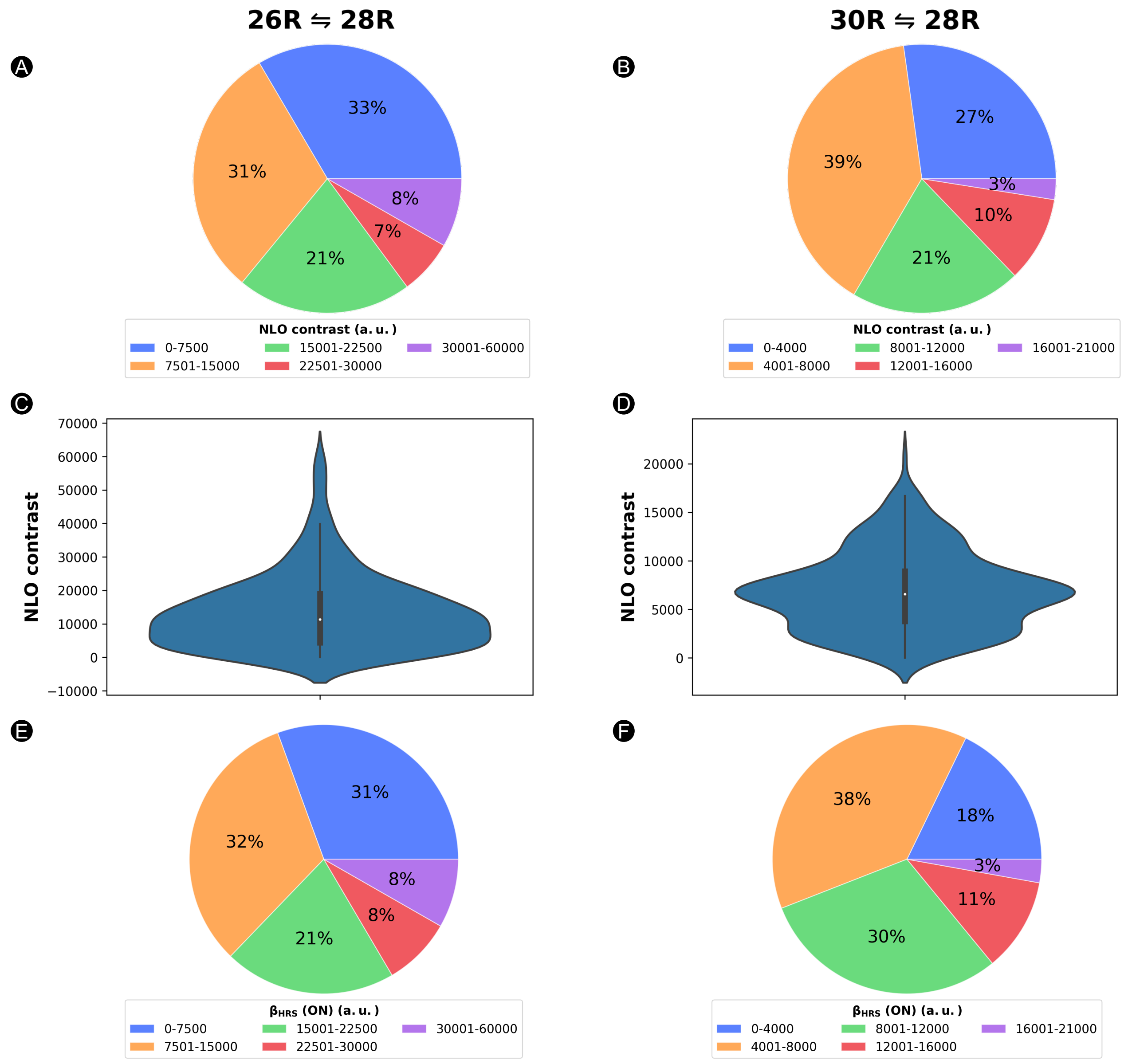

Figure S1 : (A) Pie diagram of NLO contrast of the **26R** → **28R** divided into groups ranging 7500 a.u., (B) Pie diagram of NLO contrast of the **30R** → **28R** divided into groups ranging 4000 a.u. (C) Violin plot describing the distribution of **26R** → **28R** NLO contrasts. (D) Violin plot describing the distribution of **30R** → **28R** NLO contrasts. (E) Pie diagram of β_{HRS}(ON) of the **26R** → **28R** divided into groups ranging 7500 a.u., (F) Pie diagram of β_{HRS}(ON) of the **30R** → **28R** divided into groups ranging 4000 a.u.

Figure 1C and 1D contain violin plots and boxplots representing the distribution of NLO contrasts for the **26R** and **30R**-based switches. The statistics related to the boxplot such as the quartiles (Q) and interquartile ranges (IQR) for both switches are summarized in

Table 5. The maximum of the probability distribution of the violin plot for the **30R**-based switches is located around Q2 (6578 a.u.), while the maximum for the **26R**-based switches is spread between Q1 (4385 a.u.) and Q2 (11316 a.u.). If we look for potential outliers in both datasets, we extract only 8 and 6 patterns for the **26R**- and **30R**-based switches, respectively. The largest outliers correspond to the two discussed BFS optima. Therefore, only a select group of functionalization patterns yields exceptionally large NLO contrasts.

Table S5 : Quartiles and interquartile range (IQR) of NLO contrasts (in a.u.) of the 26R and 30R-based molecular switches.

Quartiles	26R→ 28R	30R→ 28R
Q0	7	0
Q1	4385	3772
Q2	11316	6578
Q3	18968	8940
Q4	59946	20795
IQR	14583	5168
Q1 - 1.5 × IQR	-17491	-3981
Q3 + 1.5 × IQR	40843	16491

Two additional pie diagrams for the β_{HRS}(ON) responses for both redox switches are found in Figure 1E and F. The proportion of the ranges, where the β_{HRS} reaches a value above 22500 and 12000 a.u. for the **26R** → **28R** and **30R** → **28R**, respectively, remains similar to the proportions in Figure 1A and 1B with a difference of 1%, indicating that ON states with high NLO responses are generally combined with OFF states having much lower NLO responses. For the group with the lowest β_{HRS}(ON) values for the **30R** → **28R** (0-4000 a.u.), we observe a percentage decrease of 9% compared to the contrast pie diagram, which can be attributed to the coupling of ON states with noncentrosymmetric OFF states having significant enough responses. Indeed, a similar increase is observed in the range 8001-12000 a.u., showing evidence of a shift to higher β_{HRS}(ON) groups. Such dependence of the NLO contrasts on the OFF state's NLO responses is thus more prominent for the lower ON responses, whereas in the group of the best-performing NLO switches the NLO

contrast depends more on the response of the ON state than the response of the OFF state.

Since our interest lies in obtaining design rules for the best-performing hexaphyrin-based switches, we redistributed our two datasets into four groups based on their respective quartiles, namely Q0-Q1, Q1-Q2, Q2-Q3 and Q3-Q4. Our target group is primarily Q3-Q4, as this group contains the patterns with the highest NLO contrasts. In the following two subsections, we compare for each hexaphyrin redox switch which core-modifications and *meso*-substitutions are the most populated in the different groups.

4.1 Core-modifications

Figure 2 displays the percentage of core-modified structures among each quartile colored according to the number of core-modifications on sites X and/or Y.

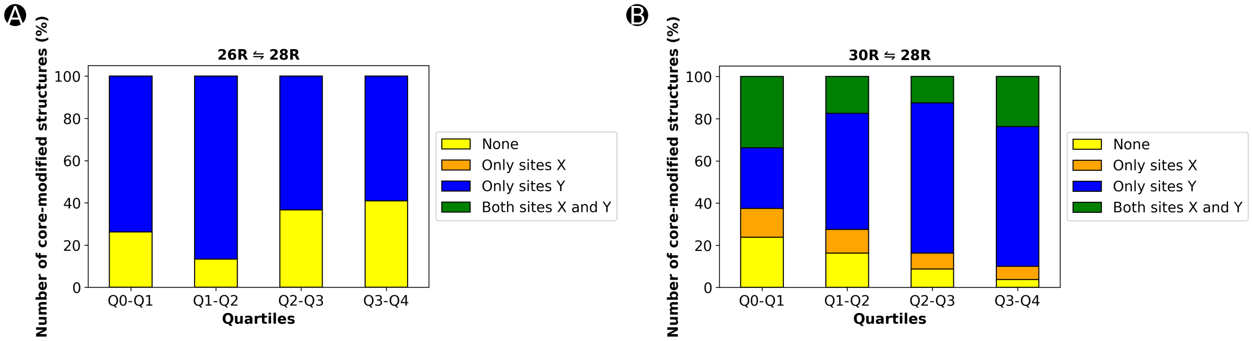

Figure S2 : Barplots of the number of core-modified structures in percentage *versus* quartiles for (A) **26R** → **28R** and (B) **30R** → **28R**.

First, no structures with core-modifications on sites X are generated for the **26R** → **28R** (Figure 2A) because this site combination was not considered during the BFS optimization procedures. In general, we observe that in our databases structures with core-modifications exceed the number of all-aza structures for both switches. For the **26R** → **28R** switches, most patterns without core-modifications are positioned in the 50% best-performing NLO switches (Q2-Q3 and Q3-Q4). By contrast, only 6.25 % of the best-performing **30R** → **28R** structures in Q2-Q3 and Q3-Q4 are not core-modified (Figure 2B), and the number

of all-aza structures increases from the higher quartiles to the lower ones representing lower NLO contrasts. As witnessed for the 50% best-performing **30R**-based switches in Figure 2B, there is a preference to modify only sites Y (69%), which is similar to the percentage (61%) for the **26R**-based switches. The presence of core-modifications on sites X, regardless of Y, is less favoured, accounting for only 32% of the total number of structures in the **30R** $\rightarrow$ **28R** dataset.

Next, we look in more detail at the type of core-modifications on these preferred sites Y. In Figure 3A, the Q2-Q3 and Q3-Q4 quartiles of the 26R database are mainly composed of structures with pyrrole rings rather than core-modified sites Y. Only in the Q2-Q3 group, a slight preference is observed for O over the other heteroatoms, when considering the top 50%. The S core-modifier dominates over all other core-modifications in Q0-Q1 and Q1-Q2, but this may be due to an increased occurrence of this type of core-modification in BFS starting structures. On the contrary, the **30R** $\rightarrow$ **28R** (Figure 3B) shows a preference for core-modifications on sites Y for the best performing group (Q3-Q4), although the favoured type of core-modification is not apparent. S is, however, clearly predominant in the second quartile Q2-Q3.

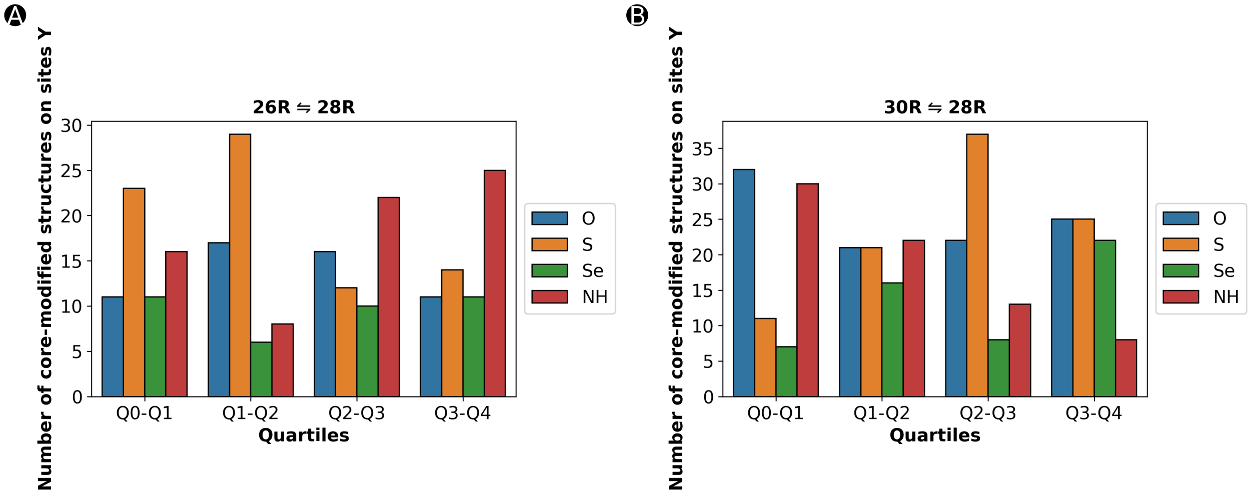

Figure S3 : Barplots of number of structures with core-modifications on sites Y colored per type and distributed within the quartiles for (A) **26R** $\rightarrow$ **28R** and (B) **30R** $\rightarrow$ **28R**.

4.2 *Meso*-substitutions

Next we focus on the *meso*-substitutions on $R_{1,4}$, $R_{2,5}$, and $R_{3,6}$. Figures 4A and 4B display the number of structures containing a combination of zero, one, two or three EDGs and/or EWGs in each quartile for **26R**- and **30R**-based switches, respectively. Mostly strong mesomerically electron-withdrawing (CN and NO_2) and electron-donating groups (OH and NH_2) are preferred by the BFS procedures to enhance the NLO contrast of hexaphyrin-based switches. In this section, we will denote these strong electron-donating groups and strong electron-withdrawing groups as EDGs and EWGs, respectively. Our main group of interest is Q3-Q4, which contains the switches with the highest NLO contrasts.

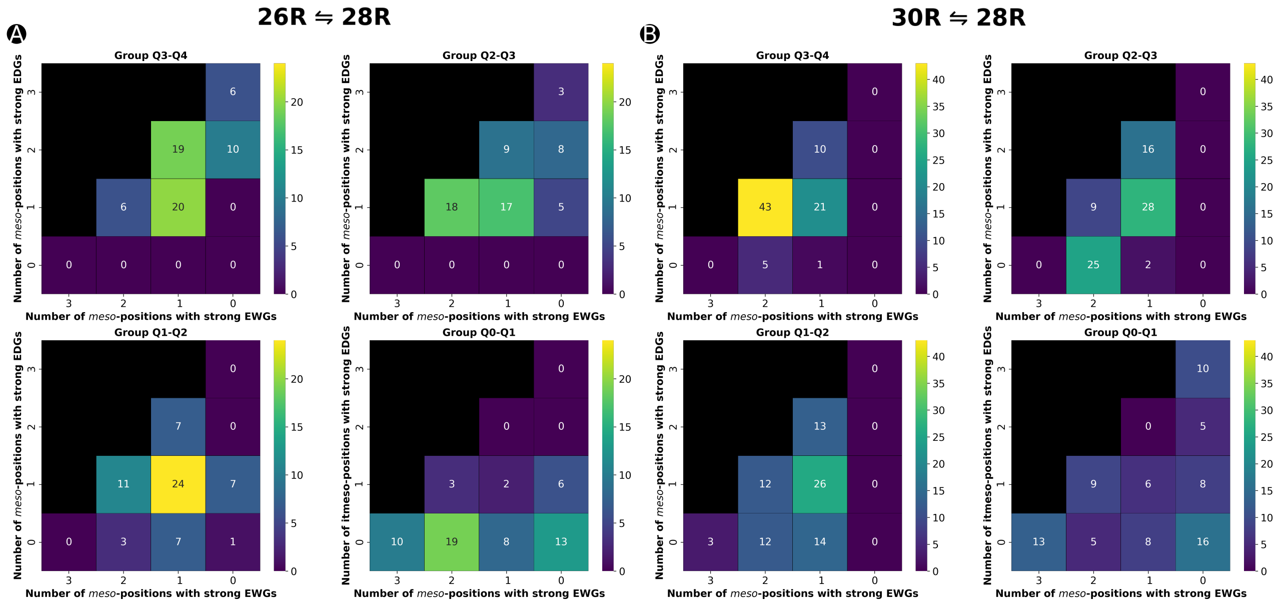

Figure S4 : Heatmap of the number of functionalized structures against the number of strongly electron-donating groups versus strongly electron-withdrawing groups on the *meso*-positions, grouped per quartile for **26R** → **28R** (A) and **30R** → **28R** (B), respectively.

For the **26R**-based switch, combinations of one or two EDGs together with one EWG are mostly present in this group. The preference for more EDGs than EWGs is also witnessed from the last column, containing 16 structures, or around 25% of the group size, having minimal 2 EDGs but no EWGs. Even more, Q3-Q4 and Q2-Q3 are not populated by structures without EDGs on the macrocyle. Note also, except for Q0-Q1, a large number

of structures with 1 EDG and 1 EWG is present in all quartiles. The *meso*-position not containing a strong EWG or EDG can either contain F, CH_3 or H. In Q1-Q2, 14 out of 24 structures have either CH_3 or F positioned on $R_{2,5}$, while 16 out of 20 structures in Q3-Q4 have these substituents positioned on all positions but $R_{2,5}$. Hence, positioning F or CH_3 on $R_{2,5}$ can significantly lower the NLO contrast. The number of structures with 2 EDGs and 1 EWG as peripheral modifications gradually decreases along the groups, going from higher to lower contrasts, hinting at their importance in the design of well-performing NLO switches. Nonetheless, structures with 2 EWGs and 1 EDG can also be found in the best half of the database, though more in the second quartile (25 – 50%). To end, the majority of the worst performing switches in group Q0-Q1 have a *meso*-substitution pattern with only EWGs and no EDGs. Hence, we can conclude that the presence of at least one EDG is crucial in enhancing the NLO contrasts of **26R**-based switches.

For the **30R**-based switches, the Q3-Q4 group consists of a combination of EWGs and EDGs, but here the largest percentage of structures contains 2 EWGs and 1 EDG, while such combinations are less populated within the best performing **26R** $\rightarrow$ **28R** switches. Q2-Q3 show a clear preference for two patterns corresponding to 1 EWG/1 EDG and 2 EWGs/0 EDG. The presence of 2 EWGs/1 EDG combinations is enormously reduced in Q2-Q3. Except for Q0-Q1, all groups have at least one set of EWGs on the macrocycle. Three patterns dominate in the low-contrast Q0-Q1 group: patterns with only EDGs, only EWGs and no EWGs/EDGs.

Therefore, both hexaphyrin-based NLO switches benefit from a combination of EDGs and EWGs to increase the NLO contrast. Nevertheless, the NLO contrast of the [26]-hexaphyrins is also enhanced with only EDGs, while this substitution pattern significantly decreases the NLO contrast of [30]-hexaphyrins. The pattern with 2 EDGs and 1 EWG is primarily favourable for the **26R** $\rightarrow$ **28R** molecular switches with 80% of such structures belonging to the Q3-Q4 and Q2-Q3 quartiles. The remaining 20% still have ON states with a considerably enhanced β_{HRS} response but they are combined with noncentrosymmetric OFF states. For

the **30R**-based molecular switches, the 2 EWGs and 2 EDGs pattern performs better with 71% in Q3-Q4 or Q2-Q3. The remaining 29% of the structures with 2 EDGs and 1 EWG are share again noncentrosymmetric OFF states, thus lowering the NLO contrasts.

In summary, two different design rules emerged from the statistical analysis of the databases generated for each redox switch. First, the **26R** → **28R** molecular switch prefers more EDGs than EWGs on their macrocycle, although also fully EDG patterns can enhance the NLO contrast. Second, the **30R** → **28R** molecular switch prefers the opposite peripheral substitution pattern with more EWGs than EDGs.

5 Collected Datasets

5.1 26R → 28R

Table S6 : Collection of the dataset generated during the BFS and prestudy containing the individual first hyperpolarizabilities of the OFF- and ON-state, NLO contrast of the 26R ⥨ 28R switch pattern. The static hyper-Rayleigh scattering first hyperpolarizability values of the [26]hexaphyrins and [28]hexaphyrins are given in a.u.

**X_Y_R$_{1,4}$_R$_{2,5}$_R$_{3,6}$**	β_{HRS}(26R)	β_{HRS}(28R)	**NLO contrast**
NH_NH_NH$_2$_CN_NH$_2$	5.99×10^4	8.19×10^{-2}	5.99×10^4
NH_Se_NH$_2$_CN_NH$_2$	5.75×10^4	1.65	5.75×10^4
NH_S_NH$_2$_CN_NH$_2$	5.50×10^4	2.66	5.50×10^4
NH_NH_NH$_2$_CN_OH	5.08×10^4	1.35×10^{-1}	5.08×10^4
NH_NH_NH$_2$_NO$_2$_NH$_2$	5.21×10^4	5.76×10^2	5.04×10^4
NH_Se_NH$_2$_CN_OH	4.33×10^4	4.74×10^{-1}	4.33×10^4
NH_S_NH$_2$_CN_OH	4.32×10^4	0.00	4.32×10^4
NH_NH_OH_CN_NH$_2$	4.28×10^4	7.67×10^{-1}	4.28×10^4
NH_NH_NH$_2$_NO$_2$_OH	4.10×10^4	3.63×10^2	3.99×10^4
NH_O_NH$_2$_CN_NH$_2$	3.84×10^4	1.12	3.84×10^4
NH_S_OH_CN_NH$_2$	3.74×10^4	4.34×10^{-1}	3.74×10^4
NH_Se_OH_CN_NH$_2$	3.89×10^4	6.43×10^2	3.7×10^4
NH_NH_NH$_2$_CN_F	3.50×10^4	7.26×10^{-1}	3.50×10^4
NH_O_NH$_2$_CN_OH	3.37×10^4	2.29	3.37×10^4
NH_NH_F_CN_NH$_2$	3.35×10^4	1.03×10^{-1}	3.35×10^4
NH_NH_NH$_2$_CH$_3$_NH$_2$	3.31×10^4	1.12×10^1	3.31×10^4
NH_NH_NH$_2$_OH_NH$_2$	3.24×10^4	1.94	3.24×10^4
NH_NH_OH_NO$_2$_NH$_2$	3.31×10^4	5.04×10^2	3.16×10^4
NH_NH_OH_OH_NH$_2$	3.10×10^4	3.65	3.10×10^4
NH_NH_NH$_2$_F_NH$_2$	3.01×10^4	2.01×10^{-1}	3.01×10^4
NH_Se_F_CN_NH$_2$	3.00×10^4	2.24	3.00×10^4
NH_Se_NH$_2$_F_NH$_2$	2.98×10^4	7.46×10^{-2}	2.98×10^4
NH_NH_NH$_2$_OH_OH	2.88×10^4	1.83	2.88×10^4
NH_S_F_CN_NH$_2$	2.87×10^4	2.56	2.87×10^4
NH_NH_NH$_2$_H_NH$_2$	2.83×10^4	8.92×10^{-1}	2.83×10^4
NH_S_NH$_2$_F_NH$_2$	2.81×10^4	7.63×10^{-1}	2.80×10^4
NH_S_OH_CN_OH	2.77×10^4	1.23×10^{-1}	2.77×10^4
NH_NH_NH$_2$_CN_CH$_3$	2.67×10^4	6.93×10^{-1}	2.67×10^4
NH_NH_NH$_2$_NO$_2$_F	2.69×10^4	1.97×10^2	2.63×10^4

Table S6 : Collection of the dataset generated during the BFS and prestudy containing the individual first hyperpolarizabilities of the OFF- and ON-state, NLO contrast of the 26R $\leftrightharpoons$ 28R switch pattern. The static hyper-Rayleigh scattering first hyperpolarizability values of the [26]hexaphyrins and [28]hexaphyrins are given in a.u.

X_Y_R$_{1,4}$_R$_{2,5}$_R$_{3,6}$	β_{HRS}(26R)	β_{HRS}(28R)	NLO contrast
NH_NH_NH$_2$_CN_H	2.53×10^4	4.92×10^{-2}	2.53×10^4
NH_S_NH$_2$_CN_F	2.53×10^4	1.03×10^1	2.53×10^4
NH_Se_NH$_2$_CN_F	2.52×10^4	8.36	2.52×10^4
NH_NH_H_CN_NH$_2$	2.47×10^4	2.07×10^{-1}	2.47×10^4
NH_NH_F_NO$_2$_NH$_2$	2.54×10^4	4.09×10^2	2.42×10^4
NH_NH_CH$_3$_CN_NH$_2$	2.41×10^4	5.00×10^{-2}	2.41×10^4
NH_NH_CN_CN_NH$_2$	2.29×10^4	7.75×10^{-1}	2.29×10^4
NH_O_NH$_2$_OH_NH$_2$	2.22×10^4	9.16×10^{-1}	2.22×10^4
NH_Se_CN_CN_NH$_2$	2.19×10^4	1.38	2.19×10^4
NH_O_OH_CN_OH	2.17×10^4	1.58	2.17×10^4
NH_O_NH$_2$_NO$_2$_NH$_2$	2.67×10^4	1.89×10^3	2.15×10^4
NH_NH_NH$_2$_OH_CN	2.14×10^4	9.94×10^{-1}	2.14×10^4
NH_S_CN_CN_NH$_2$	2.14×10^4	2.77	2.14×10^4
NH_O_NH$_2$_F_NH$_2$	2.13×10^4	2.08	2.13×10^4
NH_O_NH$_2$_CN_F	2.12×10^4	1.50	2.12×10^4
NH_Se_NO$_2$_CN_NH$_2$	2.12×10^4	1.46	2.12×10^4
NH_O_NH$_2$_OH_OH	2.12×10^4	4.93	2.12×10^4
NH_Se_H_CN_NH$_2$	2.11×10^4	3.28×10^{-2}	2.11×10^4
NH_S_NO$_2$_CN_NH$_2$	2.09×10^4	0.00	2.09×10^4
NH_NH_OH_OH_OH	2.08×10^4	2.68×10^{-1}	2.08×10^4
NH_Se_CH$_3$_CN_NH$_2$	2.08×10^4	1.15	2.08×10^4
NH_NH_NH$_2$_CN_CN	2.07×10^4	1.87×10^{-2}	2.07×10^4
NH_NH_NH$_2$_OH_F	2.05×10^4	3.23	2.05×10^4
NH_S_H_CN_NH$_2$	2.05×10^4	2.53×10^{-2}	2.05×10^4
NH_O_NH$_2$_F_OH	2.03×10^4	2.29×10^{-1}	2.03×10^4
NH_S_CH$_3$_CN_NH$_2$	2.03×10^4	1.02	2.03×10^4
NH_S_F_CN_OH	2.03×10^4	4.38×10^{-1}	2.03×10^4
NH_O_NH$_2$_CH$_3$_OH	2.03×10^4	9.39	2.02×10^4
NH_O_NH$_2$_H_NH$_2$	2.00×10^4	2.18	2.00×10^4
NH_S_NH$_2$_CN_CH$_3$	1.94×10^4	8.02	1.94×10^4
NH_S_NH$_2$_NH$_2$_CN	1.93×10^4	4.68×10^{-1}	1.93×10^4
NH_Se_NH$_2$_CN_CH$_3$	1.90×10^4	1.64×10^{-1}	1.90×10^4

Table S6 : Collection of the dataset generated during the BFS and prestudy containing the individual first hyperpolarizabilities of the OFF- and ON-state, NLO contrast of the 26R ⇋ 28R switch pattern. The static hyper-Rayleigh scattering first hyperpolarizability values of the [26]hexaphyrins and [28]hexaphyrins are given in a.u.

$X\_Y\_R_{1,4}\_R_{2,5}\_R_{3,6}$	β_{HRS}(26R)	β_{HRS}(28R)	NLO contrast
$NH\_NH\_NH_2\_NO_2\_CH$	1.94×10^4	1.68×10^2	1.89×10^4
$NH\_O\_NH_2\_H\_OH$	1.87×10^4	4.74×10^{-1}	1.87×10^4
$NH\_NH\_NH_2\_CN\_NO_2$	1.86×10^4	2.31	1.86×10^4
$NH\_S\_NH_2\_CN\_H$	1.80×10^4	4.31×10^{-2}	1.80×10^4
$NH\_NH\_H\_CN\_OH$	1.80×10^4	3.94×10^{-1}	1.80×10^4
$NH\_O\_NH_2\_NO_2\_OH$	2.41×10^4	2.36×10^3	1.79×10^4
$NH\_NH\_NH_2\_NO_2\_CH_3$	1.91×10^4	4.69×10^2	1.78×10^4
$NH\_NH\_NH_2\_OH\_CH_3$	1.76×10^4	5.84	1.76×10^4
$NH\_Se\_NH_2\_CN\_H$	1.74×10^4	2.85×10^{-2}	1.74×10^4
$NH\_NH\_H\_NO_2\_NH_2$	1.87×10^4	4.54×10^2	1.74×10^4
$NH\_NH\_OH\_OH\_CN$	1.73×10^4	1.77×10^{-1}	1.73×10^4
$NH\_NH\_F\_OH\_NH_2$	1.73×10^4	1.60	1.73×10^4
$NH\_NH\_NO_2\_CN\_NH_2$	2.43×10^4	2.92×10^3	1.68×10^4
$NH\_NH\_CN\_NO_2\_NH_2$	1.77×10^4	3.62×10^2	1.67×10^4
$NH\_O\_NH_2\_NH_2\_OH$	1.64×10^4	4.41×10^{-1}	1.64×10^4
$NH\_O\_NH_2\_CH_3\_NH_2$	1.86×10^4	8.79×10^2	1.61×10^4
$NH\_NH\_NH_2\_NO_2\_CN$	1.64×10^4	1.32×10^2	1.60×10^4
$NH\_O\_OH\_F\_NH_2$	1.59×10^4	4.30	1.59×10^4
$NH\_O\_NH_2\_NH_2\_NH_2$	1.58×10^4	1.88	1.58×10^4
$NH\_O\_NH_2\_CN\_H$	1.58×10^4	3.55×10^{-2}	1.58×10^4
$NH\_NH\_NH_2\_NH_2\_NH_2$	2.27×10^4	2.72×10^3	1.57×10^4
$NH\_Se\_NO_2\_NO_2\_NH_2$	1.63×10^4	2.13×10^2	1.57×10^4
$NH\_NH\_CN\_CN\_OH$	1.57×10^4	7.77×10^{-1}	1.57×10^4
$NH\_O\_F\_CN\_OH$	1.56×10^4	1.68×10^{-1}	1.56×10^4
$NH\_NH\_NH_2\_OH\_H$	1.53×10^4	1.09	1.53×10^4
$NH\_S\_CH_3\_CN\_OH$	1.51×10^4	6.60×10^{-2}	1.51×10^4
$NH\_S\_NH_2\_OH\_CN$	1.47×10^4	4.09×10^{-1}	1.47×10^4
$NH\_O\_NH_2\_CN\_CH_3$	1.64×10^4	6.43×10^2	1.46×10^4
$NH\_S\_H\_CN\_OH$	1.44×10^4	9.89×10^{-1}	1.44×10^4
$NH\_S\_NH_2\_CN\_CN$	1.42×10^4	1.42	1.42×10^4
$NH\_O\_CN\_CN\_NH_2$	1.42×10^4	6.57×10^{-1}	1.42×10^4
$NH\_NH\_NH_2\_NO_2\_NO_2$	1.49×10^4	2.74×10^2	1.41×10^4
$NH\_Se\_CN\_CN\_OH$	1.41×10^4	1.43	1.41×10^4
$NH\_Se\_NH_2\_CN\_CN$	1.41×10^4	1.43	1.41×10^4

Table S6 : Collection of the dataset generated during the BFS and prestudy containing the individual first hyperpolarizabilities of the OFF- and ON-state, NLO contrast of the 26R ⇋ 28R switch pattern. The static hyper-Rayleigh scattering first hyperpolarizability values of the [26]hexaphyrins and [28]hexaphyrins are given in a.u.

$X\_Y\_R_{1,4}\_R_{2,5}\_R_{3,6}$	β_{HRS}(26R)	β_{HRS}(28R)	NLO contrast
NH_S_CN_CN_OH	1.40×10^4	5.57×10^{-1}	1.40×10^4
$NH\_S\_NH_2\_CN\_NO_2$	1.36×10^4	6.73×10^{-1}	1.36×10^4
$NH\_S\_NO_2\_CN\_OH$	1.36×10^4	0.00	1.36×10^4
$NH\_Se\_NO_2\_CH_3\_NH_2$	1.35×10^4	1.85	1.35×10^4
$NH\_Se\_NO_2\_NH_2\_NH_2$	1.35×10^4	0.00	1.35×10^4
$NH\_Se\_NO_2\_CN\_OH$	1.35×10^4	0.00	1.35×10^4
$NH\_O\_NH_2\_F\_F$	1.34×10^4	8.68×10^{-2}	1.34×10^4
$NH\_NH\_NH_2\_H\_H$	1.32×10^4	4.56×10^{-1}	1.32×10^4
$NH\_Se\_NH_2\_CN\_NO_2$	1.31×10^4	2.63	1.31×10^4
$NH\_S\_NO_2\_NO_2\_NH_2$	1.60×10^4	1.14×10^3	1.29×10^4
$NH\_O\_NH_2\_CN\_CN$	1.28×10^4	6.66×10^{-1}	1.28×10^4
$NH\_S\_NO_2\_CH_3\_NH_2$	1.28×10^4	1.18	1.28×10^4
$NH\_Se\_NO_2\_OH\_NH_2$	1.26×10^4	4.75×10^{-1}	1.26×10^4
$NH\_NH\_CN\_OH\_NH_2$	1.26×10^4	1.16	1.26×10^4
$NH\_O\_NH_2\_H\_F$	1.25×10^4	1.57×10^{-2}	1.25×10^4
$NH\_NH\_H\_OH\_NH_2$	1.25×10^4	5.97×10^{-1}	1.24×10^4
$NH\_NH\_NH_2\_OH\_NO_2$	1.22×10^4	5.32×10^{-1}	1.22×10^4
$NH\_NH\_CH_3\_OH\_NH_2$	1.20×10^4	1.27	1.20×10^4
$NH\_NH\_NO_2\_OH\_NH_2$	1.20×10^4	1.81	1.20×10^4
$NH\_NH\_CH_3\_NO_2\_NH_2$	1.47×10^4	1.04×10^3	1.19×10^4
$NH\_S\_CN\_NH_2\_OH$	1.17×10^4	9.62×10^{-1}	1.17×10^4
$NH\_O\_CH_3\_CN\_OH$	1.16×10^4	3.50×10^{-1}	1.16×10^4
$NH\_Se\_NO_2\_F\_NH_2$	1.16×10^4	3.28	1.16×10^4
$NH\_O\_F\_F\_NH_2$	1.15×10^4	4.52×10^{-1}	1.15×10^4
$NH\_O\_NH_2\_F\_CH_3$	1.14×10^4	1.72×10^{-1}	1.14×10^4
NH_S_OH_CN_H	1.14×10^4	4.89	1.13×10^4
$NH\_O\_NH_2\_F\_CN$	1.13×10^4	1.17×10^{-1}	1.13×10^4
$NH\_S\_NO_2\_OH\_NH_2$	1.28×10^4	5.41×10^2	1.12×10^4
$NH\_S\_NH_2\_F\_CN$	1.12×10^4	4.57×10^{-2}	1.12×10^4
$NH\_S\_NH_2\_NO_2\_CN$	1.15×10^4	1.26×10^2	1.12×10^4
$NH\_S\_NO_2\_F\_NH_2$	1.12×10^4	2.27	1.12×10^4
NH_Se_OH_CN_H	1.11×10^4	6.75×10^{-2}	1.11×10^4
$NH\_S\_NH_2\_NO_2\_NO_2$	1.11×10^4	0.00	1.11×10^4
$NH\_NH\_H\_H\_NH_2$	1.09×10^4	1.04×10^{-1}	1.09×10^4

Table S6 : Collection of the dataset generated during the BFS and prestudy containing the individual first hyperpolarizabilities of the OFF- and ON-state, NLO contrast of the 26R ⇋ 28R switch pattern. The static hyper-Rayleigh scattering first hyperpolarizability values of the [26]hexaphyrins and [28]hexaphyrins are given in a.u.

X_Y_$R_{1,4}$_$R_{2,5}$_$R_{3,6}$	β_{HRS}(26R)	β_{HRS}(28R)	NLO contrast
NH_O_H_CN_OH	1.09×10^4	2.43×10^{-1}	1.09×10^4
NH_Se_NO_2_H_NH_2	1.07×10^4	1.50×10^{-1}	1.07×10^4
NH_S_NH_2_CH_3_CN	1.07×10^4	2.07×10^{-2}	1.07×10^4
NH_S_NO_2_NH_2_NH_2	1.06×10^4	0.00	1.06×10^4
NH_O_NH_2_H_CH_3	1.05×10^4	1.20×10^{-1}	1.05×10^4
NH_S_CN_OH_OH	1.04×10^4	5.51×10^{-1}	1.04×10^4
NH_S_NO_2_H_NH_2	1.02×10^4	3.44×10^{-1}	1.02×10^4
NH_O_NH_2_H_CN	1.02×10^4	1.54×10^{-2}	1.02×10^4
NH_NH_NO_2_NO_2_NH_2	1.82×10^4	3.42×10^3	1.01×10^4
NH_O_NH_2_F_H	1.01×10^4	1.50×10^{-2}	1.01×10^4
NH_S_NH_2_H_CN	9.99×10^3	4.00×10^{-2}	9.99×10^3
NH_S_CN_NO_2_OH	1.03×10^4	1.38×10^2	9.91×10^3
NH_O_CN_CN_OH	9.83×10^3	0.00	9.83×10^3
NH_S_CN_CH_3_OH	9.65×10^3	3.03×10^{-1}	9.65×10^3
NH_O_NH_2_H_H	9.36×10^3	1.71×10^{-2}	9.36×10^3
NH_NH_H_CN_F	9.20×10^3	0.00	9.20×10^3
NH_S_NH_2_CH_3_NO_2	8.99×10^3	1.02×10^{-1}	8.99×10^3
NH_S_NH_2_F_NO_2	8.66×10^3	2.25	8.65×10^3
NH_O_NH_2_F_NO_2	8.51×10^3	6.41	8.49×10^3
NH_S_CN_F_OH	8.40×10^3	1.01×10^{-1}	8.40×10^3
NH_S_OH_CN_CN	8.36×10^3	0.00	8.36×10^3
NH_S_NH_2_H_NO_2	8.35×10^3	1.48×10^{-1}	8.35×10^3
NH_S_NH_2_OH_NO_2	1.04×10^4	8.31×10^2	8.19×10^3
NH_O_NH_2_H_NO_2	8.09×10^3	1.03×10^1	8.06×10^3
NH_O_NO_2_CN_NH_2	1.46×10^4	2.80×10^3	8.03×10^3
NH_S_OH_CN_NO_2	7.91×10^3	3.01	7.90×10^3
NH_O_H_F_NH_2	7.77×10^3	3.69×10^{-1}	7.77×10^3
NH_O_CN_F_NH_2	7.68×10^3	1.96	7.67×10^3
NH_S_NO_2_NH_2_OH	1.02×10^4	9.73×10^2	7.63×10^3
NH_O_CH_3_F_NH_2	7.63×10^3	5.22×10^{-1}	7.63×10^3
NH_S_CN_H_OH	7.55×10^3	3.83×10^{-1}	7.55×10^3
NH_NH_F_F_F	7.52×10^3	2.58×10^{-3}	7.52×10^3
NH_NH_F_OH_CN	7.44×10^3	1.46	7.43×10^3
NH_NH_H_CN_CH_3	7.19×10^3	3.73	7.18×10^3

Table S6 : Collection of the dataset generated during the BFS and prestudy containing the individual first hyperpolarizabilities of the OFF- and ON-state, NLO contrast of the 26R $\leftrightharpoons$ 28R switch pattern. The static hyper-Rayleigh scattering first hyperpolarizability values of the [26]hexaphyrins and [28]hexaphyrins are given in a.u.

$X\_Y\_R_{1,4}\_R_{2,5}\_R_{3,6}$	β_{HRS}(26R)	β_{HRS}(28R)	NLO contrast
NH_S_NO_2_OH_OH	9.61×10^3	9.22×10^2	7.17×10^3
NH_O_OH_CN_NO_2	7.07×10^3	2.74	7.06×10^3
NH_S_NH_2_NH_2_NO_2	6.97×10^3	4.58	6.96×10^3
NH_S_F_CN_H	6.52×10^3	8.73×10^{-4}	6.52×10^3
NH_Se_F_CN_H	6.51×10^3	5.77×10^{-2}	6.51×10^3
NH_O_OH_H_H	6.11×10^3	1.64	6.10×10^3
NH_S_NO_2_CH_3_OH	9.33×10^3	1.30×10^3	6.06×10^3
NH_S_CN_CN_F	6.06×10^3	1.17	6.06×10^3
NH_Se_NO_2_CN_F	5.97×10^3	1.34	5.96×10^3
NH_O_NO_2_CN_OH	1.01×10^4	1.75×10^3	5.90×10^3
NH_NH_H_CN_H	5.73×10^3	4.62×10^{-4}	5.73×10^3
NH_S_NO_2_NO_2_OH	9.90×10^3	1.85×10^3	5.51×10^3
NH_S_NO_2_F_OH	7.97×10^3	1.02×10^3	5.37×10^3
NH_O_NO_2_F_NH_2	7.85×10^3	1.27×10^3	4.75×10^3
NH_S_CH_3_CN_H	4.69×10^3	2.65×10^{-2}	4.69×10^3
NH_Se_CH_3_CN_H	4.68×10^3	8.38×10^{-2}	4.68×10^3
NH_NH_H_OH_CN	4.65×10^3	6.66×10^{-1}	4.64×10^3
NH_Se_NO_2_CN_CH_3	4.41×10^3	0.00	4.41×10^3
NH_S_CN_CN_CH_3	4.38×10^3	6.49×10^{-1}	4.38×10^3
NH_S_NO_2_H_OH	7.15×10^3	1.14×10^3	4.35×10^3
NH_S_F_CN_NO_2	4.21×10^3	2.70	4.20×10^3
NH_S_NO_2_CN_F	5.90×10^3	6.73×10^2	4.16×10^3
NH_S_F_CN_CN	4.08×10^3	1.66	4.07×10^3
NH_O_NH_2_CN_NO_2	1.18×10^4	3.96×10^3	3.89×10^3
NH_S_H_CN_H	3.87×10^3	0.00	3.87×10^3
NH_Se_H_CN_H	3.85×10^3	2.82×10^{-2}	3.85×10^3
NH_Se_H_NH_2_H	3.70×10^3	2.12×10^{-1}	3.70×10^3
NH_NH_H_CH_3_H	3.47×10^3	1.76×10^{-1}	3.47×10^3
NH_O_F_CN_NO_2	3.44×10^3	0.00	3.44×10^3
NH_S_H_NH_2_H	3.35×10^3	1.58×10^{-1}	3.35×10^3
NH_NH_H_CN_NO_2	3.33×10^3	5.51×10^{-1}	3.33×10^3
NH_NH_H_CN_CN	3.12×10^3	1.00×10^{-1}	3.12×10^3
NH_Se_CN_CN_H	3.03×10^3	1.47×10^{-2}	3.03×10^3

Table S6 : Collection of the dataset generated during the BFS and prestudy containing the individual first hyperpolarizabilities of the OFF- and ON-state, NLO contrast of the 26R $\leftrightharpoons$ 28R switch pattern. The static hyper-Rayleigh scattering first hyperpolarizability values of the [26]hexaphyrins and [28]hexaphyrins are given in a.u.

$X\_Y\_R_{1,4}\_R_{2,5}\_R_{3,6}$	β_{HRS}(26R)	β_{HRS}(28R)	NLO contrast
NH_S_CN_CN_H	2.97×10^3	2.83×10^{-2}	2.97×10^3
NH_Se_NO_2_CN_H	2.97×10^3	2.53×10^{-2}	2.97×10^3
NH_NH_H_OH_H	2.91×10^3	1.55	2.91×10^3
NH_Se_H_OH_H	2.80×10^3	9.66×10^{-1}	2.80×10^3
NH_S_H_OH_H	2.80×10^3	2.21	2.80×10^3
NH_S_CH_3_CN_NO_2	2.78×10^3	5.73	2.76×10^3
NH_NH_CN_OH_CN	4.10×10^3	5.28×10^2	2.76×10^3
NH_O_F_H_H	2.72×10^3	0.00	2.72×10^3
NH_NH_H_F_H	2.61×10^3	2.93×10^{-3}	2.61×10^3
NH_NH_CH_3_OH_CN	4.77×10^3	9.16×10^2	2.61×10^3
NH_NH_H_NH_2_H	2.49×10^3	1.64	2.48×10^3
NH_NH_H_NO_2_H	3.35×10^3	3.35×10^2	2.47×10^3
NH_S_CH_3_CN_CN	2.36×10^3	2.15×10^{-1}	2.36×10^3
NH_Se_H_CH_3_H	2.12×10^3	1.77×10^{-1}	2.12×10^3
NH_S_H_CH_3_H	2.11×10^3	1.48×10^{-1}	2.11×10^3
NH_S_H_NO_2_H	2.64×10^3	2.01×10^2	2.09×10^3
NH_Se_H_NO_2_H	2.73×10^3	2.41×10^2	2.09×10^3
NH_NH_H_H_H	2.09×10^3	5.04×10^{-3}	2.09×10^3
NH_S_H_CN_NO_2	1.97×10^3	1.51	1.97×10^3
NH_Se_H_F_H	1.82×10^3	1.64×10^{-2}	1.82×10^3
NH_S_H_F_H	1.78×10^3	0.00	1.78×10^3
NH_NH_NO_2_OH_N	4.07×10^3	1.11×10^3	1.70×10^3
NH_O_H_CN_NO_2	1.60×10^3	0.00	1.60×10^3
NH_O_CH_3_H_H	1.55×10^3	3.34×10^{-1}	1.55×10^3
NH_S_H_CN_CN	1.52×10^3	7.42×10^{-2}	1.52×10^3
NH_S_NO_2_CN_H	2.98×10^3	6.50×10^2	1.49×10^3
NH_S_CN_CN_NO_2	1.46×10^3	8.96×10^{-1}	1.46×10^3
NH_Se_H_H_H	1.45×10^3	3.80	1.44×10^3
NH_S_H_H_H	1.37×10^3	2.25	1.36×10^3
NH_NH_NO_2_CN_H	4.52×10^3	1.64×10^3	1.35×10^3
NH_O_CN_H_H	1.16×10^3	6.78×10^4	1.16×10^3

Table S6 : Collection of the dataset generated during the BFS and prestudy containing the individual first hyperpolarizabilities of the OFF- and ON-state, NLO contrast of the 26R ⇆ 28R switch pattern. The static hyper-Rayleigh scattering first hyperpolarizability values of the [26]hexaphyrins and [28]hexaphyrins are given in a.u.

$X\_Y\_R_{1,4}\_R_{2,5}\_R_{3,6}$	β_{HRS}(26R)	β_{HRS}(28R)	NLO contrast
$NH\_S\_NO_2\_CN\_CH_3$	4.37×10^3	1.72×10^3	1.16×10^3
NH_O_H_H_H	1.01×10^3	5.67×10^{-1}	1.01×10^3
$NH\_Se\_NO_2\_CN\_CN$	9.83×10^2	0.00	9.83×10^2
$NH\_NH\_CH_3\_CH_3\_CH_3$	3.51×10^3	1.52×10^3	7.89×10^2
NH_S_CN_CN_CN	7.35×10^2	6.06×10^{-1}	7.33×10^2
$NH\_Se\_NO_2\_CN\_NO_2$	1.36×10^3	6.32×10^2	2.65×10^2
$NH\_O\_NO_2\_CN\_NO_2$	1.27×10^3	5.89×10^2	2.52×10^2
$NH\_S\_NO_2\_CN\_NO_2$	1.29×10^3	6.26×10^2	2.29×10^2
$NH\_O\_CN\_CN\_NO_2$	1.38×10^3	7.66×10^2	1.78×10^2
$NH\_NH\_NO_2\_NO_2\_NO_2$	1.36×10^3	8.78×10^2	1.05×10^2
$NH\_O\_CH_3\_CN\_NO_2$	2.17×10^3	1.60×10^3	8.81×10^1
$NH\_S\_NO_2\_CN\_CN$	8.24×10^2	1.08×10^3	3.56×10^1
NH_NH_CN_CN_CN	1.41×10^3	1.16×10^3	2.44×10^1
$NH\_NH\_NO_2\_H\_H$	1.11×10^3	1.26×10^3	9.52
$NH\_O\_NO_2\_H\_H$	7.12×10^2	8.17×10^2	7.18

5.2 30R → 28R

Table S7 : Collection of the dataset generated during the BFS and prestudy containing the individual first hyperpolarizabilities of the OFF- and ON-state, NLO contrast of the 30R ⇆ 28R switch pattern. The static hyper-Rayleigh scattering first hyperpolarizability values of the [30]hexaphyrins and [28]hexaphyrins are given in a.u.

**X_Y_R$_{1,4}$_R$_{2,5}$_R$_{3,6}$**	β_{HRS}(30R)	β_{HRS}(28R)	**NLO contrast**
O_O_NH$_2$_CN_CN	2.08×10^4	6.18×10^{-1}	2.08×10^4
O_S_NH$_2$_CN_CN	1.72×10^4	3.36	1.72×10^4
O_O_NH$_2$_CN_NO$_2$	1.71×10^4	0.00	1.71×10^4
NH_O_NH$_2$_CN_CN	1.70×10^4	6.66×10^{-1}	1.70×10^4
NH_S_NO$_2$_CN_NH$_2$	1.67×10^4	0.00	1.67×10^4
O_O_NH$_2$_NO$_2$_CN	1.72×10^4	1.98×10^2	1.66×10^4
NH_Se_NO$_2$_CN_NH$_2$	1.61×10^4	1.46	1.61×10^4
O_O_OH_CN_CN	1.61×10^4	1.19	1.61×10^4
O_NH_CN_CN_NH$_2$	1.58×10^4	1.57	1.58×10^4
O_NH_NH$_2$_CN_CN	1.57×10^4	2.09	1.57×10^4
NH_O_OH_CN_NO$_2$	1.55×10^4	2.74	1.55×10^4
O_S_CN_CN_NH$_2$	1.52×10^4	0.00	1.52×10^4
O_O_OH_CN_NO$_2$	1.46×10^4	0.00	1.46×10^4
NH_S_NO$_2$_H_NH$_2$	1.45×10^4	3.44×10^{-1}	1.45×10^4
NH_S_NO$_2$_NH$_2$_NH$_2$	1.44×10^4	0.00	1.44×10^4
NH_O_OH_CN_CN	1.42×10^4	2.67	1.42×10^4
O_O_CN_CN_NH$_2$	1.40×10^4	1.73	1.40×10^4
NH_O_OH_NH$_2$_NO$_2$	1.39×10^4	0.00	1.39×10^4
NH_Se_NO$_2$_OH_NH$_2$	1.39×10^4	4.75×10^{-1}	1.39×10^4
NH_S_CN_CN_NH$_2$	1.36×10^4	2.77	1.36×10^4
NH_S_NH$_2$_CN_CN	1.35×10^4	1.42	1.35×10^4
NH_S_NH$_2$_CN_NO$_2$	1.35×10^4	6.73×10^{-1}	1.35×10^4
NH_O_OH_H_NO$_2$	1.35×10^4	2.05	1.35×10^4
O_Se_NH$_2$_CN_CN	1.34×10^4	8.47×10^{-1}	1.34×10^4
NH_S_NO$_2$_F_NH$_2$	1.34×10^4	2.27	1.34×10^4
NH_S_NO$_2$_CH$_3$_NH$_2$	1.34×10^4	1.18	1.34×10^4
NH_S_NO$_2$_OH_NH$_2$	1.46×10^4	5.41×10^2	1.31×10^4
NH_O_F_CN_NO$_2$	1.31×10^4	0.00	1.31×10^4
O_Se_CN_CN_NH$_2$	1.31×10^4	1.65	1.31×10^4
O_S_NO$_2$_CN_NH$_2$	1.38×10^4	2.96×10^2	1.29×10^4
NH_Se_CN_CN_NH$_2$	1.29×10^4	1.38	1.29×10^4

Table S7 : Collection of the dataset generated during the BFS and prestudy containing the individual first hyperpolarizabilities of the OFF- and ON-state, NLO contrast of the 30R ⇋ 28R switch pattern. The static hyper-Rayleigh scattering first hyperpolarizability values of the [30]hexaphyrins and [28]hexaphyrins are given in a.u.

**X_Y_R$_{1,4}$_R$_{2,5}$_R$_{3,6}$**	β_{HRS}(30R)	β_{HRS}(28R)	**NLO contrast**
NH_S_NO_2_CN_OH	1.28×10^4	0.00	1.28×10^4
NH_Se_NO_2_NO_2_NH_2	1.32×10^4	2.13×10^2	1.26×10^4
NH_NH_CN_CN_NH_2	1.25×10^4	7.75×10^{-1}	1.25×10^4
NH_S_CN_CN_OH	1.25×10^4	5.57×10^{-1}	1.25×10^4
NH_O_OH_F_NO_2	1.25×10^4	9.01×10^{-1}	1.25×10^4
O_Se_NO_2_CN_NH_2	1.23×10^4	0.00	1.23×10^4
O_NH_CN_CN_OH	1.22×10^4	1.78	1.22×10^4
NH_S_OH_CN_CN	1.20×10^4	0.00	1.20×10^4
NH_Se_NO_2_H_NH_2	1.20×10^4	1.50×10^{-1}	1.20×10^4
NH_O_CN_CN_NH_2	1.20×10^4	6.57×10^{-1}	1.20×10^4
NH_Se_NH_2_CN_NO_2	1.20×10^4	2.63	1.20×10^4
NH_O_H_CN_NO_2	1.19×10^4	0.00	1.19×10^4
NH_Se_NO_2_CN_OH	1.18×10^4	0.00	1.18×10^4
NH_Se_CN_CN_OH	1.17×10^4	1.43	1.17×10^4
S_O_NH_2_CN_CN	1.71×10^4	2.13×10^3	1.17×10^4
NH_NH_NH_2_CN_CN	1.15×10^4	1.87×10^{-2}	1.15×10^4
NH_S_CN_NO_2_OH	1.19×10^4	1.38×10^2	1.14×10^4
NH_Se_NH_2_CN_CN	1.14×10^4	1.43	1.14×10^4
NH_S_OH_CN_NO_2	1.14×10^4	3.01	1.14×10^4
NH_Se_NO_2_NH_2_NH_2	1.13×10^4	0.00	1.13×10^4
NH_S_NO_2_NO_2_NH_2	1.44×10^4	1.14×10^3	1.13×10^4
O_Se_NH_2_CN_NO_2	1.12×10^4	1.06	1.12×10^4
NH_O_OH_OH_NO_2	1.11×10^4	2.08	1.11×10^4
NH_S_NH_2_NH_2_NO_2	1.11×10^4	4.58	1.11×10^4
NH_NH_CN_CN_OH	1.09×10^4	7.77×10^{-1}	1.09×10^4
NH_Se_NO_2_F_NH_2	1.09×10^4	3.28	1.09×10^4
NH_Se_NO_2_CH_3_NH_2	1.08×10^4	1.85	1.08×10^4
O_O_NH_2_CN_H	1.07×10^4	1.29×10^{-1}	1.07×10^4
O_O_NH_2_H_CN	1.06×10^4	2.20×10^{-2}	1.06×10^4
NH_S_NH_2_CH_3_NO_2	1.06×10^4	1.02×10^{-1}	1.06×10^4
NH_Se_NH_2_OH_NO_2	1.04×10^4	0.00	1.04×10^4
NH_Se_NH_2_NH_2_NO_2	1.04×10^4	4.36	1.03×10^4
NH_S_NH_2_H_NO_2	1.00×10^4	1.48×10^{-1}	1.00×10^4

Table S7 : Collection of the dataset generated during the BFS and prestudy containing the individual first hyperpolarizabilities of the OFF- and ON-state, NLO contrast of the 30R ⇋ 28R switch pattern. The static hyper-Rayleigh scattering first hyperpolarizability values of the [30]hexaphyrins and [28]hexaphyrins are given in a.u.

$X\_Y\_R_{1,4}\_R_{2,5}\_R_{3,6}$	β_{HRS}(30R)	β_{HRS}(28R)	NLO contrast
$O\_O\_NH_2\_F\_CN$	1.00×10^4	1.19	1.00×10^4
$O\_O\_NH_2\_CH_3\_CN$	9.86×10^3	1.18	9.86×10^3
$NH\_S\_F\_CN\_NO_2$	9.75×10^3	2.70	9.74×10^3
$NH\_O\_CN\_CN\_OH$	9.71×10^3	0.00	9.71×10^3
$NH\_S\_NH_2\_F\_NO_2$	9.70×10^3	2.25	9.69×10^3
$NH\_Se\_NH_2\_CH_3\_NO_2$	9.58×10^3	2.88×10^{-1}	9.58×10^3
$NH\_Se\_NO_2\_CN\_F$	9.56×10^3	1.34	9.56×10^3
$O\_NH\_NH_2\_H\_CN$	9.51×10^3	1.67×10^{-2}	9.51×10^3
$NH\_Se\_OH\_CN\_NO_2$	9.49×10^3	7.66×10^{-1}	9.49×10^3
$NH\_S\_NH_2\_OH\_NO_2$	1.17×10^4	8.31×10^2	9.40×10^3
$NH\_O\_H\_H\_NO_2$	9.30×10^3	1.01	9.30×10^3
$NH\_S\_CN\_H\_OH$	9.11×10^3	3.83×10^{-1}	9.11×10^3
$NH\_O\_NH_2\_H\_CN$	9.07×10^3	1.54×10^{-2}	9.07×10^3
$O\_O\_F\_CN\_CN$	9.04×10^3	8.06×10^{-1}	9.04×10^3
$O\_NH\_CN\_H\_NH_2$	9.00×10^3	0.00	9.00×10^3
$NH\_Se\_NH_2\_H\_NO_2$	8.95×10^3	6.77×10^{-1}	8.94×10^3
$O\_O\_NH_2\_CN\_F$	8.94×10^3	1.31	8.94×10^3
$NH\_S\_CN\_NH_2\_OH$	8.93×10^3	9.62×10^{-1}	8.93×10^3
$NH\_NH\_NH_2\_CN\_NO_2$	8.92×10^3	2.31	8.91×10^3
$NH\_S\_F\_CN\_CN$	8.89×10^3	1.66	8.89×10^3
$S\_S\_NH_2\_CN\_CN$	1.20×10^4	1.18×10^3	8.87×10^3
$O\_O\_NH_2\_CN\_CH_3$	8.84×10^3	2.63×10^{-1}	8.84×10^3
$NH\_S\_NO_2\_CN\_F$	1.07×10^4	6.73×10^2	8.83×10^3
$NH\_S\_CN\_CN\_F$	8.80×10^3	1.17	8.80×10^3
$NH\_S\_CN\_CH_3\_OH$	8.79×10^3	3.03×10^{-1}	8.79×10^3
$NH\_S\_CH_3\_CN\_NO_2$	8.80×10^3	5.73	8.78×10^3
$NH\_S\_NH_2\_CH_3\_CN$	8.68×10^3	2.07×10^{-2}	8.68×10^3
$NH\_Se\_NH_2\_F\_NO_2$	8.64×10^3	2.27	8.63×10^3
$NH\_S\_NH_2\_NH_2\_CN$	8.59×10^3	4.68×10^{-1}	8.59×10^3
$Se\_O\_NH_2\_CN\_CN$	1.39×10^4	2.17×10^3	8.55×10^3
$O\_NH\_H\_CN\_NH_2$	8.55×10^3	4.15	8.54×10^3
$NH\_S\_CN\_OH\_OH$	8.51×10^3	5.51×10^{-1}	8.50×10^3
$O\_NH\_NO_2\_CN\_NH_2$	1.02×10^4	6.01×10^2	8.50×10^3

Table S7 : Collection of the dataset generated during the BFS and prestudy containing the individual first hyperpolarizabilities of the OFF- and ON-state, NLO contrast of the 30R $\leftrightharpoons$ 28R switch pattern. The static hyper-Rayleigh scattering first hyperpolarizability values of the [30]hexaphyrins and [28]hexaphyrins are given in a.u.

X_Y_$R_{1,4}$_$R_{2,5}$_$R_{3,6}$	β_{HRS}(30R)	β_{HRS}(28R)	NLO contrast
NH_O_CN_CN_F	8.36×10^3	8.95×10^{-1}	8.36×10^3
NH_S_NO_2_OH_OH	1.08×10^4	9.22×10^2	8.35×10^3
NH_S_CN_F_OH	8.33×10^3	1.01×10^{-1}	8.33×10^3
NH_S_H_CN_NO_2	8.23×10^3	1.51	8.23×10^3
NH_Se_NO_2_CN_CH_3	8.22×10^3	0.00	8.22×10^3
NH_S_CH_3_CN_CN	8.22×10^3	2.15×10^{-1}	8.22×10^3
NH_O_CN_H_NH_2	8.15×10^3	1.60	8.15×10^3
NH_S_NH_2_H_CN	8.11×10^3	4.00×10^{-2}	8.11×10^3
NH_S_NO_2_H_OH	1.11×10^4	1.14×10^3	8.08×10^3
O_O_CH_3_CN_CN	8.08×10^3	1.78×10^{-1}	8.07×10^3
NH_S_CN_NO_2_F	8.44×10^3	1.54×10^2	7.99×10^3
NH_O_NH_2_CN_NH_2	7.94×10^3	1.12	7.93×10^3
NH_S_CN_CN_CH_3	7.80×10^3	6.49×10^{-1}	7.79×10^3
NH_S_NH_2_CN_NH_2	7.80×10^3	2.66	7.79×10^3
NH_Se_NO_2_CN_H	7.78×10^3	2.53×10^{-2}	7.78×10^3
NH_O_H_OH_NO_2	7.70×10^3	8.51×10^{-1}	7.70×10^3
NH_O_CN_F_NH_2	7.69×10^3	1.96	7.68×10^3
NH_O_CN_CH_3_NH_2	7.85×10^3	5.49×10^1	7.68×10^3
NH_S_NH_2_F_CN	7.67×10^3	4.57×10^{-2}	7.67×10^3
NH_O_OH_CN_OH	7.50×10^3	1.58	7.50×10^3
O_O_NH_2_OH_CN	7.49×10^3	2.31×10^{-1}	7.49×10^3
NH_S_NH_2_OH_CN	7.48×10^3	4.09×10^{-1}	7.48×10^3
NH_NH_H_CN_NO_2	7.48×10^3	5.51×10^{-1}	7.47×10^3
NH_O_OH_CN_H	7.47×10^3	2.62	7.46×10^3
NH_NH_NH_2_H_CN	7.45×10^3	2.34×10^{-2}	7.45×10^3
NH_S_NO_2_NH_2_OH	1.00×10^4	9.73×10^2	7.44×10^3
NH_O_CN_CN_CH_3	7.44×10^3	3.61×10^{-1}	7.44×10^3
NH_Se_F_CN_NO_2	7.42×10^3	7.92	7.39×10^3
NH_S_OH_CN_NH_2	7.32×10^3	4.34×10^{-1}	7.32×10^3
NH_S_NO_2_F_OH	1.00×10^4	1.02×10^3	7.32×10^3
NH_S_NO_2_NO_2_OH	1.19×10^4	1.85×10^3	7.31×10^3
NH_O_OH_CN_CH_3	7.24×10^3	1.12	7.24×10^3
NH_O_OH_CN_NH_2	7.22×10^3	1.07	7.22×10^3

Table S7 : Collection of the dataset generated during the BFS and prestudy containing the individual first hyperpolarizabilities of the OFF- and ON-state, NLO contrast of the 30R ⇋ 28R switch pattern. The static hyper-Rayleigh scattering first hyperpolarizability values of the [30]hexaphyrins and [28]hexaphyrins are given in a.u.

$\mathbf{X\_Y\_R_{1,4}\_R_{2,5}\_R_{3,6}}$	β_{HRS}(30R)	β_{HRS}(28R)	NLO contrast
NH_Se_CH_3_CN_NO_2	7.20×10^3	5.50×10^{-1}	7.20×10^3
NH_S_NO_2_CN_H	8.94×10^3	6.50×10^2	7.17×10^3
O_O_H_CN_CN	7.16×10^3	8.95×10^{-2}	7.16×10^3
Se_S_NH_2_CN_CN	1.10×10^4	1.54×10^3	7.13×10^3
NH_Se_NH_2_CN_NH_2	7.11×10^3	1.65	7.11×10^3
NH_S_NO_2_CH_3_OH	1.04×10^4	1.30×10^3	7.09×10^3
NH_NH_OH_CN_NO_2	7.06×10^3	0.00	7.06×10^3
NH_S_NO_2_NO_2_F	9.17×10^3	7.94×10^2	7.04×10^3
O_NH_CH_3_CN_NH_2	7.03×10^3	1.07	7.02×10^3
O_NH_CN_NO_2_NH_2	7.57×10^3	1.94×10^2	7.01×10^3
NH_O_CN_OH_NH_2	8.67×10^3	6.11×10^2	7.00×10^3
O_O_NH_2_NH_2_CN	7.89×10^3	3.20×10^2	6.98×10^3
NH_Se_CN_NO_2_F	7.47×10^3	1.78×10^2	6.96×10^3
O_NH_F_CN_NH_2	6.95×10^3	2.83	6.95×10^3
NH_S_H_CN_NH_2	6.95×10^3	2.53×10^{-2}	6.95×10^3
NH_O_CN_CN_H	6.92×10^3	7.81×10^{-1}	6.92×10^3
S_S_CN_CN_NH_2	1.08×10^4	1.59×10^3	6.84×10^3
NH_S_CN_CN_H	6.82×10^3	2.83×10^{-2}	6.82×10^3
NH_O_H_F_NO_2	8.41×10^3	5.88×10^2	6.80×10^3
O_NH_CN_CN_F	6.80×10^3	7.11×10^{-1}	6.80×10^3
NH_S_H_CN_CN	6.77×10^3	7.42×10^{-2}	6.77×10^3
NH_S_CH_3_CN_NH_2	6.77×10^3	1.02	6.76×10^3
NH_NH_H_CN_NH_2	6.76×10^3	2.07×10^{-1}	6.76×10^3
NH_NH_NH_2_CN_NH_2	6.75×10^3	8.19×10^{-1}	6.75×10^3
NH_S_H_CN_OH	6.75×10^3	9.89×10^{-1}	6.75×10^3
NH_NH_H_CN_CN	6.72×10^3	1.00×10^{-1}	6.72×10^3
NH_Se_H_CN_NH_2	6.71×10^3	3.28×10^{-2}	6.71×10^3
NH_O_OH_CN_F	6.71×10^3	1.85×10^1	6.66×10^3
NH_S_CH_3_CN_OH	6.62×10^3	6.60×10^{-2}	6.62×10^3
NH_S_CN_H_F	6.59×10^3	1.10×10^{-2}	6.59×10^3
O_NH_NH_2_CN_NH_2	6.58×10^3	2.18	6.57×10^3
NH_S_F_CN_NH_2	6.57×10^3	2.56	6.56×10^3
NH_O_CN_NO_2_F	8.09×10^3	5.56×10^2	6.56×10^3

Table S7 : Collection of the dataset generated during the BFS and prestudy containing the individual first hyperpolarizabilities of the OFF- and ON-state, NLO contrast of the 30R $\leftrightharpoons$ 28R switch pattern. The static hyper-Rayleigh scattering first hyperpolarizability values of the [30]hexaphyrins and [28]hexaphyrins are given in a.u.

**X_Y_R$_{1,4}$_R$_{2,5}$_R$_{3,6}$**	β_{HRS}(30R)	β_{HRS}(28R)	**NLO contrast**
NH_S_OH_CN_OH	6.54×10^3	1.23×10^{-1}	6.54×10^3
O_Se_NH$_2$_F_NO$_2$	6.55×10^3	3.39	6.54×10^3
NH_S_CN_NH$_2$_F	6.53×10^3	1.00	6.53×10^3
NH_S_NH$_2$_CN_H	6.52×10^3	4.31×10^{-2}	6.52×10^3
NH_NH_CH$_3$_CN_NH$_2$	6.51×10^3	5.00×10^{-2}	6.51×10^3
NH_O_NO$_2$_OH_NH$_2$	9.37×10^3	1.11×10^3	6.51×10^3
NH_Se_CH$_3$_CN_NH$_2$	6.51×10^3	1.15	6.51×10^3
NH_S_NH$_2$_CN_OH	6.50×10^3	0.00	6.50×10^3
NH_NH_H_H_NO$_2$	6.41×10^3	4.23	6.4×10^3
NH_S_NH$_2$_NO$_2$_NO$_2$	6.40×10^3	0.00	6.40×10^3
O_NH_CN_CN_CH$_3$	6.36×10^3	5.12×10^{-1}	6.36×10^3
O_O_NH$_2$_CN_NH$_2$	7.47×10^3	3.99×10^2	6.36×10^3
NH_S_CN_CH$_3$_F	6.36×10^3	6.03×10^{-1}	6.36×10^3
NH_S_NH$_2$_CN_CH$_3$	6.37×10^3	8.02	6.34×10^3
NH_NH_OH_CN_NH$_2$	6.32×10^3	7.67×10^{-1}	6.31×10^3
NH_NH_F_CN_NH$_2$	6.29×10^3	1.03×10^{-1}	6.29×10^3
NH_O_F_CN_NH$_2$	6.25×10^3	1.33	6.24×10^3
NH_S_NH$_2$_CN_F	6.27×10^3	1.03×10^1	6.24×10^3
NH_Se_F_CN_NH$_2$	6.23×10^3	2.24	6.23×10^3
NH_Se_H_CN_NO$_2$	6.20×10^3	3.27×10^{-1}	6.20×10^3
NH_O_H_CN_NH$_2$	6.18×10^3	1.58	6.17×10^3
NH_O_H_NH$_2$_NO$_2$	9.02×10^3	1.12×10^3	6.16×10^3
O_O_H_CN_NO$_2$	6.13×10^3	0.00	6.13×10^3
NH_O_CH$_3$_CN_NH$_2$	6.10×10^3	9.78×10^{-1}	6.10×10^3
NH_NH_H_CN_OH	6.08×10^3	3.94×10^{-1}	6.08×10^3
NH_Se_NH$_2$_CN_OH	6.07×10^3	4.74×10^{-1}	6.06×10^3
NH_S_F_CN_OH	6.05×10^3	4.38×10^{-1}	6.04×10^3
NH_S_NH$_2$_NO$_2$_CN	6.40×10^3	1.26×10^2	6.03×10^3
NH_O_H_H_CN	5.97×10^3	7.07×10^{-1}	5.97×10^3
NH_O_OH_CH$_3$_NO$_2$	1.12×10^4	2.23×10^3	5.97×10^3
NH_S_CN_F_F	5.95×10^3	2.17	5.95×10^3
NH_O_OH_NO$_2$_NO$_2$	5.86×10^3	1.37×10^{-1}	5.85×10^3
NH_Se_NH$_2$_CN_H	5.81×10^3	2.85×10^{-2}	5.81×10^3

Table S7 : Collection of the dataset generated during the BFS and prestudy containing the individual first hyperpolarizabilities of the OFF- and ON-state, NLO contrast of the 30R ⇋ 28R switch pattern. The static hyper-Rayleigh scattering first hyperpolarizability values of the [30]hexaphyrins and [28]hexaphyrins are given in a.u.

$X\_Y\_R_{1,4}\_R_{2,5}\_R_{3,6}$	β_{HRS}(30R)	β_{HRS}(28R)	NLO contrast
S_O_H_CN_NO_2	7.34×10^3	5.88×10^2	5.75×10^3
NH_Se_NH_2_CN_CH_3	5.67×10^3	1.64×10^{-1}	5.67×10^3
NH_Se_NH_2_NO_2_NO_2	6.20×10^3	1.86×10^2	5.67×10^3
NH_Se_NH_2_CN_F	5.68×10^3	8.36	5.65×10^3
O_O_NH_2_CN_OH	6.43×10^3	2.79×10^2	5.64×10^3
O_NH_CN_F_NH_2	8.43×10^3	1.12×10^3	5.59×10^3
O_NH_CN_OH_NH_2	8.46×10^3	1.15×10^3	5.57×10^3
NH_S_H_H_NO_2	5.51×10^3	1.09	5.50×10^3
NH_O_CN_NH_2_NH_2	8.38×10^3	1.14×10^3	5.49×10^3
NH_O_H_CH_3_NO_2	8.00×10^3	1.03×10^3	5.38×10^3
NH_NH_CN_NO_2_F	5.98×10^3	2.57×10^2	5.26×10^3
NH_Se_OH_CN_NH_2	6.90×10^3	6.43×10^2	5.19×10^3
NH_S_NH_2_NO_2_F	5.61×10^3	1.46×10^2	5.19×10^3
O_NH_CN_CH_3_NH_2	8.65×10^3	1.44×10^3	5.15×10^3
O_NH_CN_NH_2_NH_2	8.03×10^3	1.16×10^3	5.14×10^3
O_NH_CN_CN_H	5.11×10^3	8.95×10^{-1}	5.11×10^3
Se_S_CN_CN_NH_2	9.62×10^3	1.96×10^3	5.06×10^3
S_Se_NH_2_CN_NO_2	6.76×10^3	6.85×10^2	4.96×10^3
O_NH_OH_CN_NH_2	5.53×10^3	2.02×10^2	4.95×10^3
NH_S_NO_2_CN_CH_3	8.99×10^3	1.72×10^3	4.94×10^3
NH_NH_CN_H_H	4.83×10^3	5.15×10^{-1}	4.83×10^3
NH_NH_H_H_CN	4.83×10^3	4.05×10^{-1}	4.83×10^3
NH_Se_H_H_NO_2	4.66×10^3	2.00	4.66×10^3
O_NH_CN_NO_2_F	5.20×10^3	2.29×10^2	4.56×10^3
NH_S_CN_OH_F	6.41×10^3	7.18×10^2	4.54×10^3
NH_NH_H_CN_F	4.53×10^3	0.00	4.53×10^3
Se_O_H_CN_NO_2	6.33×10^3	7.12×10^2	4.48×10^3
NH_NH_H_CN_CH_3	4.40×10^3	3.73	4.39×10^3
NH_O_NH_2_CN_NO_2	1.24×10^4	3.96×10^3	4.34×10^3
NH_S_CN_CN_NO_2	4.33×10^3	8.96×10^{-1}	4.33×10^3
NH_NH_NO_2_CN_H	8.12×10^3	1.64×10^3	4.31×10^3
NH_Se_CN_CN_NO_2	4.30×10^3	8.32×10^{-1}	4.30×10^3
S_Se_NO_2_CN_NH_2	1.01×10^4	2.78×10^3	4.13×10^3

Table S7 : Collection of the dataset generated during the BFS and prestudy containing the individual first hyperpolarizabilities of the OFF- and ON-state, NLO contrast of the 30R ⇋ 28R switch pattern. The static hyper-Rayleigh scattering first hyperpolarizability values of the [30]hexaphyrins and [28]hexaphyrins are given in a.u.

**X_Y_R$_{1,4}$_R$_{2,5}$_R$_{3,6}$**	β_{HRS}**(30R)**	β_{HRS}**(28R)**	**NLO contrast**
S_O_CN_CN_NH_2	8.94×10^3	2.17×10^3	4.12×10^3
S_Se_NH_2_F_NO_2	4.08×10^3	7.95×10^{-1}	4.08×10^3
NH_Se_NO_2_CN_CN	4.03×10^3	0.00	4.03×10^3
S_S_NO_2_CN_NH_2	1.00×10^4	2.84×10^3	4.01×10^3
NH_S_CH_3_NO_2_F	4.40×10^3	1.66×10^2	3.93×10^3
NH_NH_H_CN_H	3.88×10^3	4.62×10^{-4}	3.88×10^3
S_O_NH_2_F_NO_2	1.06×10^4	3.27×10^3	3.88×10^3
Se_O_CN_CN_NH_2	8.96×10^3	2.36×10^3	3.85×10^3
NH_O_CH_3_CN_NO_2	7.47×10^3	1.60×10^3	3.81×10^3
NH_S_H_CN_H	3.79×10^3	0.00	3.79×10^3
NH_NH_NO_2_CN_NH_2	9.86×10^3	2.92×10^3	3.77×10^3
Se_Se_NH_2_F_NO_2	3.77×10^3	0.00	3.77×10^3
O_NH_H_H_CN	3.70×10^3	2.81×10^{-1}	3.70×10^3
NH_O_CN_CN_CN	3.55×10^3	5.66×10^{-1}	3.55×10^3
NH_NH_H_H_NH_2	3.49×10^3	1.04×10^{-1}	3.49×10^3
NH_NH_NH_2_H_H	3.49×10^3	4.56×10^{-1}	3.49×10^3
NH_NH_NO_2_H_H	6.42×10^3	1.26×10^3	3.47×10^3
S_O_OH_CN_NO_2	6.94×10^3	1.57×10^3	3.38×10^3
O_NH_CN_CN_NO_2	3.38×10^3	2.43	3.37×10^3
NH_S_F_NO_2_F	4.14×10^3	2.78×10^2	3.37×10^3
NH_S_CN_CN_CN	3.31×10^3	6.06×10^{-1}	3.31×10^3
S_O_NH_2_F_CN	7.56×10^3	1.99×10^3	3.25×10^3
Se_Se_NO_2_CN_NH_2	9.27×10^3	3.00×10^3	3.20×10^3
O_NH_CN_CN_CN	3.13×10^3	5.68×10^{-1}	3.13×10^3
O_O_CN_CN_CN	3.07×10^3	1.00	3.07×10^3
NH_S_H_NO_2_F	3.74×10^3	2.65×10^2	3.01×10^3
Se_S_NO_2_CN_NH_2	9.04×10^3	3.03×10^3	2.99×10^3
NH_NH_H_H_OH	2.96×10^3	9.93×10^{-1}	2.96×10^3
NH_S_NO_2_CN_NO_2	4.38×10^3	6.26×10^2	2.81×10^3
Se_O_OH_CN_NO_2	6.44×10^3	1.67×10^3	2.80×10^3
S_NH_CN_CN_NH_2	9.30×10^3	3.47×10^3	2.66×10^3
NH_NH_H_H_F	2.62×10^3	3.24	2.61×10^3
NH_NH_NH_2_OH_NH_2	2.60×10^3	1.94	2.60×10^3

Table S7 : Collection of the dataset generated during the BFS and prestudy containing the individual first hyperpolarizabilities of the OFF- and ON-state, NLO contrast of the 30R $\leftrightharpoons$ 28R switch pattern. The static hyper-Rayleigh scattering first hyperpolarizability values of the [30]hexaphyrins and [28]hexaphyrins are given in a.u.

**X_Y_$R_{1,4}$_$R_{2,5}$_$R_{3,6}$**	β_{HRS}(30R)	β_{HRS}(28R)	NLO contrast
NH_O_NO_2_CN_NO_2	4.05×10^3	5.89×10^2	2.58×10^3
NH_NH_H_H_CH_3	2.53×10^3	1.32	2.53×10^3
NH_O_H_OH_NH_2	2.48×10^3	9.88×10^{-1}	2.48×10^3
NH_O_H_H_H	2.47×10^3	5.67×10^{-1}	2.47×10^3
NH_Se_NO_2_CN_NO_2	3.99×10^3	6.32×10^2	2.44×10^3
S_O_H_F_NO_2	5.06×10^3	1.23×10^3	2.33×10^3
S_O_F_F_NO_2	5.79×10^3	1.63×10^3	2.33×10^3
O_O_NO_2_CN_CN	3.18×10^3	3.38×10^2	2.30×10^3
NH_NH_H_NO_2_H	3.16×10^3	3.35×10^2	2.28×10^3
NH_O_CN_CN_NO_2	4.04×10^3	7.66×10^2	2.23×10^3
NH_O_H_NO_2_NO_2	5.02×10^3	1.29×10^3	2.21×10^3
NH_O_CH_3_OH_NH_2	2.20×10^3	8.50×10^{-1}	2.19×10^3
NH_NH_H_H_H	2.18×10^3	5.04×10^{-3}	2.18×10^3
Se_NH_H_H_H	2.17×10^3	5.72×10^{-3}	2.17×10^3
S_O_OH_F_NO_2	6.98×10^3	2.47×10^3	2.15×10^3
NH_O_NO_2_CN_NH_2	7.47×10^3	2.80×10^3	2.12×10^3
O_NH_H_H_H	2.09×10^3	1.09×10^{-2}	2.09×10^3
S_NH_H_H_H	2.06×10^3	2.82×10^{-1}	2.06×10^3
Se_Se_H_H_H	2.05×10^3	3.07×10^{-1}	2.05×10^3
O_O_H_H_H	2.03×10^3	1.52	2.03×10^3
NH_S_H_H_H	2.02×10^3	2.25	2.01×10^3
NH_O_F_OH_NH_2	2.01×10^3	2.75	2.00×10^3
NH_O_NH_2_OH_NH_2	2.00×10^3	9.16×10^{-1}	2.00×10^3
Se_NH_CN_CN_NH_2	7.92×10^3	3.23×10^3	1.97×10^3
S_O_CH_3_F_NO_2	5.39×10^3	1.67×10^3	1.97×10^3
S_S_H_H_H	1.96×10^3	7.24×10^{-1}	1.96×10^3
S_NH_CN_NO_2_F	4.74×10^3	1.32×10^3	1.93×10^3
NH_O_CN_NO_2_NH_2	4.70×10^3	1.31×10^3	1.91×10^3
NH_Se_H_H_H	1.92×10^3	3.80	1.91×10^3
NH_NH_F_F_F	1.87×10^3	2.58×10^{-1}	1.87×10^3
Se_Se_NH_2_CN_NO_2	5.76×10^3	2.00×10^3	1.82×10^3
NH_Se_NH_2_OH_NH_2	3.67×10^3	8.24×10^2	1.81×10^3

Table S7 : Collection of the dataset generated during the BFS and prestudy containing the individual first hyperpolarizabilities of the OFF- and ON-state, NLO contrast of the 30R ⇋ 28R switch pattern. The static hyper-Rayleigh scattering first hyperpolarizability values of the [30]hexaphyrins and [28]hexaphyrins are given in a.u.

X_Y_$R_{1,4}$_$R_{2,5}$_$R_{3,6}$	β_{HRS}(30R)	β_{HRS}(28R)	NLO contrast
NH_NH_H_CH_3_H	1.79×10^3	1.76×10^{-1}	1.79×10^3
NH_S_NO_2_CN_CN	4.15×10^3	1.08×10^3	1.79×10^3
NH_S_OH_NO_2_F	4.97×10^3	1.56×10^3	1.77×10^3
Se_NH_CN_NO_2_F	4.92×10^3	1.6×10^3	1.70×10^3
NH_NH_OH_OH_OH	1.67×10^3	2.68×10^{-1}	1.67×10^3
NH_NH_H_F_H	1.61×10^3	2.93×10^{-3}	1.61×10^3
NH_S_NH_2_OH_NH_2	3.27×10^3	7.67×10^2	1.55×10^3
NH_NH_H_OH_H	1.35×10^3	1.55	1.35×10^3
NH_NH_H_NH_2_H	1.32×10^3	1.64	1.31×10^3
NH_NH_NO_2_NO_2_NO_2	3.18×10^3	8.78×10^2	1.30×10^3
NH_NH_CN_CN_CN	3.52×10^3	1.16×10^3	1.19×10^3
S_O_CN_F_NO_2	3.68×10^3	1.42×10^3	1.00×10^3
S_O_NH_2_F_H	3.46×10^3	1.35×10^3	9.22×10^2
NH_O_OH_OH_NH_2	2.33×10^3	7.64×10^2	7.94×10^2
S_O_NH_2_F_F	2.98×10^3	1.24×10^3	7.16×10^2
S_S_NH_2_F_NO_2	4.66×10^3	2.44×10^3	6.94×10^2
S_O_NH_2_F_CH_3	3.14×10^3	1.55×10^3	5.38×10^2
S_O_NO_2_F_NO_2	2.86×10^3	1.36×10^3	5.31×10^2
S_O_NH_2_F_NH_2	2.65×10^3	1.57×10^3	2.76×10^2
S_O_NH_2_F_OH	2.47×10^3	1.47×10^3	2.53×10^2
S_O_NH_2_OH_NH_2	1.71×10^3	2.60×10^3	1.84×10^2
NH_NH_CH_3_CH_3_CH_3	2.19×10^3	1.52×10^3	1.22×10^2
Se_O_NH_2_OH_NH_2	2.35×10^3	2.85×10^3	4.65×10^1
O_O_NH_2_OH_NH_2	2.21×10^3	2.55×10^3	2.40×10^1
NH_NH_NH_2_NH_2_NH_2	2.88×10^3	2.72×10^3	5.11
S_NH_NH_2_F_NO_2	2.91×10^3	2.89×10^3	7.20×10^{-1}

References

(1) Frisch, M. J.; Trucks, G. W.; Schlegel, H. B.; Scuseria, G. E.; Robb, M. A.; Cheeseman, J. R.; Scalmani, G.; Barone, V.; Petersson, G. A.; Nakatsuji, H. *et al.* Gaussian 16 Revision A.01. 2016; Gaussian Inc. Wallingford CT.

(2) Yanai, T.; Tew, D. P.; Handy, N. C. A new hybrid exchange–correlation functional using the coulomb-attenuating method (CAM-B3LYP). *Chem. Phys. Lett.* **2004**, *393*, 51 – 57.

(3) Hehre, W. J.; Radom, L.; Schleyer, P. v. R.; Pople, J. A. *Ab initio molecular orbital theory*; Wiley, 1986.

(4) Torrent-Sucarrat, M.; Navarro, S.; Cossío, F. P.; Anglada, J. M.; Luis, J. M. Relevance of the DFT method to study expanded porphyrins with different topologies. *J. Comput. Chem.* **2017**, *38*, 2819–2828.

(5) Woller, T.; Banerjee, A.; Sylvetsky, N.; Santra, G.; Deraet, X.; De Proft, F.; Martin, J. M. L.; Alonso, M. Performance of electronic structure methods for the description of Hückel–Möbius interconversions in extended π-systems. *J. Phys. Chem. A* **2020**, *124*, 2380–2397.

(6) Sylvetsky, N.; Banerjee, A.; Alonso, M.; Martin, J. M. L. Performance of localized coupled cluster methods in a moderately strong correlation regime: Hückel–Möbius interconversions in expanded porphyrins. *J. Chem. Theory Comput.* **2020**, *16*, 3641–3653.

(7) Torrent-Sucarrat, M.; Anglada, J. M.; Luis, J. M. Evaluation of the nonlinear optical properties for an expanded porphyrin Hückel-Möbius aromaticity switch. *J. Chem. Phys.* **2012**, *137*, 184306.

(8) Torrent-Sucarrat, M.; Anglada, J. M.; Luis, J. M. Evaluation of the nonlinear optical properties for annulenes with Hückel and Möbius topologies. *J. Chem. Theory Comput.* **2011**, *7*, 3935–3943.

(9) Torrent-Sucarrat, M.; Navarro, S.; Marcos, E.; Anglada, J. M.; Luis, J. M. Design of Hückel–Möbius topological switches with high nonlinear optical properties. *J. Phys. Chem. C.* **2017**, *121*, 19348–19357.

(10) Castet, F.; Rodriguez, V.; Pozzo, J.-L.; Ducasse, L.; Plaquet, A.; Champagne, B. Design and characterization of molecular nonlinear optical switches. *Acc. Chem. Res.* **2013**, *46*, 2656–2665.

(11) Plaquet, A.; Guillaume, M.; Champagne, B.; Castet, F.; Ducasse, L.; Pozzo, J.-L.; Rodriguez, V. In silico optimization of merocyanine-spiropyran compounds as second-order nonlinear optical molecular switches. *Phys. Chem. Chem. Phys.* **2008**, *10*, 6223–6232.

(12) de Wergifosse, M.; Champagne, B. Electron correlation effects on the first hyperpolarizability of push–pull π-conjugated systems. *J. Chem. Phys.* **2011**, *134*, 074113.

(13) Lescos, L.; Sitkiewicz, S. P.; Beaujean, P.; Blanchard-Desce, M.; Champagne, B.; Matito, E.; Castet, F. Performance of DFT functionals for calculating the second-order nonlinear optical properties of dipolar merocyanines. *Phys. Chem. Chem. Phys.* **2020**, *22*, 16579–16594.

(14) Teunissen, J. L. Inverse molecular design: optimization and application of combinatorial and stochastic approaches. Ph.D. thesis, Vrije Universteit Brussel, 2019.

(15) Teunissen, J. L. CINDES. https://gitlab.com/jlteunissen/CINDES, [Accessed on October 1, 2023].

(16) RDKit: Open-source cheminformatics. http://www.rdkit.org, [Accessed on September 7, 2023].

(17) Rogers, D.; Hahn, M. Extended-Connectivity Fingerprints. *J. Chem. Inf. Model.* **2010**, *50*, 742–754.

(18) Cihan Sorkun, M.; Mullaj, D.; Koelman, J. M. V. A.; Er, S. ChemPlot, a python library for chemical space visualization. *Chemistry–Methods* **2022**, *2*, e202200005.

(19) van der Maaten, L.; Hinton, G. Visualizing high-dimensional data Using t-SNE. *J. Mach. Learn. Res.* **2008**, *9*, 2579–2605.

(20) Pedregosa, F.; Varoquaux, G.; Gramfort, A.; Michel, V.; Thirion, B.; Grisel, O.; Blondel, M.; Prettenhofer, P.; Weiss, R.; Dubourg, V. *et al.* Scikit-learn: machine learning in python. *J. Mach. Learn. Res.* **2011**, *12*, 2825–2830.

(21) Desmedt, E.; Woller, T.; Teunissen, J. L.; De Vleeschouwer, F.; Alonso, M. Fine-tuning of nonlinear optical contrasts of hexaphyrin-based molecular switches using inverse design. *Front. Chem.* **2021**, *9*, 786036.

(22) Desmedt, E.; Smets, D.; Woller, T.; Alonso, M.; De Vleeschouwer, F. Designing hexaphyrins for high-potential NLO switches: the synergy of core-modifications and *meso*-substitutions. *Phys. Chem. Chem. Phys.* **2023**, *25*, 17128–17142.
